# The Phosphofructokinase Isoform AtPFK5 Is a Novel Target of Plastidic Thioredoxin-f-Dependent Redox Regulation

**DOI:** 10.3390/antiox10030401

**Published:** 2021-03-07

**Authors:** Natalia Hess, Simon Richter, Michael Liebthal, Karl-Josef Dietz, Angelika Mustroph

**Affiliations:** 1 Plant Physiology, University Bayreuth, Universitaetsstr. 30, 95440 Bayreuth, Germany; natalia.h1@gmx.de (N.H.); Simon.Richter@uni-bayreuth.de (S.R.); 2 Department of Biochemistry and Physiology of Plants, University of Bielefeld, 33615 Bielefeld, Germany; mliebthal@uni-bielefeld.de (M.L.); karl-josef.dietz@uni-bielefeld.de (K.-J.D.)

**Keywords:** phosphofructokinase, thioredoxin, *Arabidopsis thaliana*, redox regulation

## Abstract

The chloroplast primary metabolism is of central importance for plant growth and performance. Therefore, it is tightly regulated in order to adequately respond to multiple environmental conditions. A major fluctuation that plants experience each day is the change between day and night, i.e., the change between assimilation and dissimilation. Among other mechanisms, thioredoxin-mediated redox regulation is an important component of the regulation of plastid-localized metabolic enzymes. While assimilatory processes such as the Calvin–Benson cycle are activated under illumination, i.e., under reducing conditions, carbohydrate degradation is switched off during the day. Previous analyses have identified enzymes of the oxidative pentose phosphate pathway to be inactivated by reduction through thioredoxins. In this work, we present evidence that an enzyme of the plastidic glycolysis, the phosphofructokinase isoform AtPFK5, is also inactivated through reduction by thioredoxins, namely by thioredoxin-f. With the help of chemical oxidation, mutant analyses and further experiments, the highly conserved motif CXDXXC in AtPFK5 was identified as the target sequence for this regulatory mechanism. However, knocking out this isoform in plants had only very mild effects on plant growth and performance, indicating that the complex primary metabolism in plants can overcome a lack in AtPFK5 activity.

## 1. Introduction

Thiol redox regulation is an important mechanism of post-translational protein modifications in plant responses to changing environmental conditions. This regulatory mechanism affects virtually all processes in plant cells ranging from, e.g., transcription, translation, metabolism, to cell division and cell fate. One major function is the switch between catabolic and anabolic pathways, namely between activation of the oxidative and reductive pentose phosphate pathways in darkness and light, respectively (summarized in [1]). Light-driven photosynthetic electron transport (PET) reduces ferredoxin (FDX) which in turn provides electrons for reduction of thioredoxin (TRX) through FDX-dependent TRX reductase (FTR) at two neighboring Cys residues (Cys), thereby reducing the disulfide bond to dithiols. This reducing power is then transferred to target enzymes also containing neighboring Cys (summarized in [2,3]). Depending on the nature of the target enzyme, the reduction of oxidized half-cystine residues can either activate or deactivate the enzyme activity.

PET builds-up reducing and phosphorylation power as well as regulatory intermediates, which altogether activate specific enzymes and enable carbon assimilation. Enzymes of the reductive pentose phosphate pathway (i.e., the Calvin-Benson cycle) are activated by reduction, for example Rubisco activase, phosphoglycerate kinase, 3-phosphoglyceraldehyde dehydrogenase, triosephosphate isomerase and fructose-1,6-bisphosphatase [4,5]. This is achieved through TRX-f and -m (summarized in [6]). In parallel, the breakdown of carbohydrates within chloroplasts through the oxidative pentose phosphate pathway is switched-off in the light and activated in darkness. Rate-controlling enzymes of the oxidative pentose phosphate pathway are deactivated by reduction, namely glucose-6-phosphate dehydrogenase (G6PDH) [5] and potentially 6-phosphogluconate dehydrogenase [5,7,8]. G6PDH is the best-analyzed protein so far which is inactivated in light [9,10,11]. This redox regulatory system utilizing a large variety of TRXs thus prevents a futile cycle of catabolic and anabolic reactions at the same time.

TRX-mediated redox regulation balances electron provision by PET and consumption of reducing power and energy in the Calvin-Benson cycle and other metabolic pathways. Additionally, TRX controls mechanisms to avoid over-reduction of the system. One of those target enzymes is the plastidic malate dehydrogenase (NADP-MDH), whose function is the formation of malate under oxidation of NADPH to regenerate NADP^+^ as final electron acceptor of PET. Malate is then transferred into the cytosol to remove excessive reducing power from the chloroplast [12]. NADP-MDH is preferentially activated by reduction through the TRX isoforms TRX-f and TRX-m (summarized in [2,6,13]).

Besides the availability of reducing power through the PET, the sugar status of a plant cell can also mediate redox regulations. In the plastid, this is mediated by NADP-TRX reductase C (NTRC) which controls, among others, the activity of ADP-glucose pyrophosphorylase (e.g., [14,15,16]).

Once reduced, mechanisms must be at hand to oxidize the thiols in order to achieve efficient thiol switching. The fast re-oxidation of Cys at the target enzymes upon transfer from light to dark is mediated by TRX and TRX-like proteins and thiol peroxidases such as peroxiredoxins [17,18,19]. This mechanism links production of H_2_O_2_ and thiol redox regulation in chloroplasts in context of photosynthesis and may serve as blueprint for similar regulatory scenarios [3].

Experimental evidence suggests that many more TRX target proteins exist besides the established classical enzymes. Proteomic approaches have identified extended sets of potential targets (e.g., [7,8,20,21,22], summarized in [5,13,23]. Likely, other enzymes in primary metabolism are also regulated through redox regulation. For example, cytosolic aldolase is also a TRX target [24].

This work aimed to explore the possibility that the ATP-dependent phosphofructokinase (PFK) is subject to thiol-redox regulation. This glycolysis-specific enzyme forms a small protein family and catalyzes the irreversible phosphorylation of fructose-6-phosphate (Fru6P) to fructose-1,6-bisphosphate (Fru1,6BP). Earlier studies reported redox-sensitivity of its activity [25,26,27]. However, redox regulation requires reversibility by reduction, e.g., by TRXs [5,23].

In *Arabidopsis thaliana*, the PFK family consists of seven isoforms, of which two are localized in plastids and five in the cytosol [28,29]. This gene family consists of three subgroups, of which subgroup PFK_A contains mainly cytosolic isoforms, and subgroup PFK_C the major plastidic isoforms (Supplementary Figure S1 and [30]). The importance of the catalyzed reaction in central carbon metabolism [31] and its irreversibility suggest that PFK could be a target of tight regulation. Furthermore, plastid enzymes are often regulated by redox regulation, including enzymes of the Calvin-Benson cycle and the oxidative pentose phosphate pathway. In this work, we identified the plastid-localized isoform AtPFK5 of subgroup PFK_C as a redox-regulated enzyme which is deactivated upon reduction and consistently switched off in the light. This mechanism, together with the inactivation of the oxidative pentose phosphate pathway, prevents the degradation of hexose phosphates in the chloroplast during the day. Very recently, another group has uncovered the same mechanism through independent techniques, and thus confirms our findings [32].

## 2. Materials and Methods

### 2.1. Plant Material, Growth Conditions and Treatment

Tobacco (*Nicotiana benthamiana*) plants were grown in soil in a growth chamber under long-day conditions with a 16-h photoperiod and a photon flux density of 85 µmol photons m^−2^ s^−1^. The temperature was maintained at 24 °C. 6-week-old plants were infiltrated with Agrobacteria containing *A. thaliana* phosphofructokinase sequences.

Arabidopsis wildtype Col-0, *pfk4* and *pfk5* mutants (*pfk4*, SALK_012602; *pfk5-1*, SALK_067384; *pfk5-2*, SAIL_694_A07 [33]) were ordered from NASC, and the insertion site of the T-DNA was verified by PCR on genomic DNA as well as by sequencing of the PCR products (Supplementary Figure S2). For *AtPFK5* transcript analysis, RNA isolation from plant tissue and cDNA synthesis was described previously in detail [34]. RT-PCR was performed using primers listed in Supplementary Table S1. The double mutant *pfk4 pfk5-2* was obtained by crossing and subsequent genotyping of the F2 and F3 generation.

For enzyme assays and genotyping, seeds were surface-sterilized, plated onto vertical plates containing Murashige and Skoog (MS) medium (Duchefa, Haarlem, The Netherlands) with 1% (*w*/*v*) sucrose and 1% (*w*/*v*) agar), stratified for 3 days in darkness at 4 °C). Subsequently, the plates were grown for 7 days in phytotrons under long-day (LD) conditions (23 °C, 16 h/8 h light/dark cycle; 100 µmol of photons m^−2^ sec^−1^). For experiments with adult plants, 7-day-old seedlings were transplanted into soil and grown under the same conditions as tobacco plants (see above) for 4 weeks.

For root growth assays on vertical plates, the growth medium was based on 1/10 strength modified Hoagland’s solution previously described [35] with 1% (*w*/*v*) sucrose, 0.05% (*w*/*v*) 2-(*N*-morpho-lino) ethanesulfonic acid (pH 5.7), and 1% (*w*/*v*) agar type E (Sigma-Aldrich, Taufkirchen, Germany). Fe was supplied as N,N′-Di-(2-hydroxybenzoyl)-ethylenediamine-N,N′-diacetic acid (Fe-HBED) to a final concentration of 5 μM. After stratification for 2 days at 4 °C seedlings were incubated under long-day conditions (16 h light/8 h dark) at 22 °C with 130 µmol photons m^−2^ s^−1^. Plant root length was measured with a ruler after 14 days of growth.

### 2.2. Creation of CRISPR/Cas9 Knockout Lines for AtPFK5

In order to generate independent knockout lines for *AtPFK5*, the CRISPR/Cas9 toolkit from Xing et al. [36] was used. Primers for cloning of gRNAs (Supplementary Table S1) were designed at two different position of the *AtPFK5* gene (Supplementary Figure S2A), and cloned into pKSE401 [36]. The constructs were verified by sequencing and subsequently transferred into Agrobacteria, which were used for floral-dip mediated transformation of *A. thaliana* Col-0. Leaves of T1 plants were checked for occurrence of mutations by PCR on genomic DNA and subsequent sequencing of the PCR product using primers spanning the CRISPR site (Supplementary Table S1). In subsequent generations, mutant lines were isolated that were homozygous for the mutation and had lost the transgene containing the CRISPR/Cas9 T-DNA insertion.

### 2.3. Cultivation of Trypanosoma Brucei Brucei

Procyclic *Trypanosoma brucei brucei* wildtype strain 427 was grown on SDM-79 standard medium with 23.8 mM sodium hydrogen carbonate, 11.5 mM hemin and 10 % *v*/*v* fetal bovine serum (FCS) at a pH of 7.3. Cultivation took place at 27 °C until cells reached a density of 2 × 10^7^.

### 2.4. Agrobacterium tumefaciens-Mediated Transient Gene Expression in Nicotiana benthamiana Plants

Transient transformation of *Nicotiana benthamiana* was performed according to Bendahmane et al. [37]. The *Agrobacterium tumefaciens* strain LBD4404 harboring PFK cDNA-containing constructs was cultivated overnight in 50 mL YEB medium containing the selection markers kanamycin (50 µg/mL) and rifampicin (50 µg/mL), 1 mM MES, pH 5.2 and 20 μM acetosyringone. Cells were pelleted by centrifugation at 3500× *g* and 4 °C for 20 min and washed afterwards with water. After a second centrifugation step, the pellet was re-suspended in infiltration buffer containing 10 mM MgCl_2_, 10 mM MES, pH 5.2, and 0.1 mM acetosyringone and adjusted to a final OD_600_ of 1.0. After incubation for 2 h at room temperature, the suspension was infiltrated at the lower side of a *Nicotiana benthamiana* leaf using a 1 mL blunt end tip syringe. Plants were grown for three more days, and subsequently leaf tissue was harvested for further analyses.

### 2.5. Cloning and Site-Directed Mutagenesis of PFKs for Transient Overexpression in Nicotiana benthamiana

The PFK constructs have been described previously [28]. *AtPFK5* was newly cloned according to the previous procedure by use of the new reverse primer 5′-TATTCTAGATTAGATGAAATCGGGTTGGCCC-3′. Plant RNA from 7-day-old seedlings was isolated with TRIsure (Bioline GmbH, Luckenwalde, Germany), according to the protocol, and cDNA was made by use of RevertAid Reverse Transcriptase (Lifetechnologies, Darmstadt, Germany). The PCR product was cloned into the vector pBinAR as previously described [28].

For site-directed mutagenesis of *AtPFK3* and *AtPFK5*, the method described by Zheng et al. [38] was used. The *AtPFK3* and *AtPFK5* coding sequences (CDS) were amplified by use of the primers described in Supplementary Table S1 and subsequently cloned into the vector pGEM-T (Promega, Mannheim, Germany). Oligonucleotides (Supplementary Table S1) containing the corresponding point mutation were used for the amplification of the whole vector. The newly amplified vector lacked *E. coli* strain DH10β-based DNA methylation. In order to separate old vector without mutation, DpnI endonuclease restriction of the PCR product was performed. An aliquot of the restriction digestion was transformed into *E. coli*, clones were selected and point mutations were verified by sequencing. Correct *AtPFK3* and *AtPFK5* clones with point mutations were cloned into the pBinAR according to the cloning strategy used before [28].

### 2.6. Preparation of Chloroplast Extracts and Treatment

*Nicotiana benthamiana* leaves were infiltrated with *AtPFK5*, *AtPFK4* or infiltration buffer. Three days after infiltration, leaves were mechanically homogenized into crude pieces in a chloroplast extraction buffer containing 0.4 M sucrose, 20 mM tricine and 10 mM NaCl (pH 8.0 with NaOH). The homogenate that contained intact chloroplasts was filtered through gauze tissues to separate debris from the clear extract. One part of the chloroplast suspension was illuminated for up to one hour at 1000 µmol photons m^−2^ s^−1^, the other half was incubated in the dark. Subsequently, intact chloroplasts from the extract were pelleted at 1800× *g* for 2 min and frozen in liquid nitrogen. Afterward enzymes were extracted and PFK activity was determined as mentioned below. Chloroplasts from *A. thaliana* rosette leaves were isolated by use of the same procedure.

### 2.7. Enzyme Analysis

Protein from leaf discs that were ground in liquid nitrogen, or from frozen chloroplasts were extracted in 50 mM HEPES-KOH, pH 6.8, containing 5 mM magnesium acetate, 5 mM β-mercaptoethanol, 15% (*v*/*v*) glycerol, 1 mM EDTA, 1 mM EGTA, 5 mM DTT and 0.1 mM Pefabloc proteinase inhibitor (Sigma Aldrich, Steinheim, Germany). The homogenate was centrifuged at 13,000× *g* at 4 °C for 20 min. The supernatant was used for spectrophotometric determination of the activities of the PFKs at 340 nm using a Specord 200 photometer (Analytik Jena, Jena, Germany).

PFK activity was measured under standard and partly denaturing conditions. The activity of PFK (EC 2.7.1.11) under standard conditions was assayed in a reaction buffer containing 0.1 M HEPES-KOH, pH 7.9, 2 mM MgCl_2_, 0.15 mM NADH, 7.5 mM Fru-6-P, 1 U aldolase, 1 U triosephosphate isomerase and 1 U glycerol-3-phosphate dehydrogenase [28]. The reaction was started by adding ATP to a final concentration of 2.5 mM. For assaying PFK activity under oxidizing conditions, the enzyme extract was oxidized with 2.5 mM sodium tetrathionate and incubated at room temperature for 20 min. An aliquot was used for measuring the PFK activity, the remaining enzyme extract was again re-reduced with 5 mM DTT. For determination of PFK activity under partly denaturing conditions the enzyme extract was incubated for 20 min with 175 µM Bis-Tris propane (pH 9.0) and 500 mM NaCl [39]. For reduction 10 mM DTT was added. The protein content was measured according to Bradford [40] and calculated based on a calibration curve using BSA as standard.

For treatment of PFK and MDH with TRX, the enzyme extract was prepared as described above, and was subsequently preincubated with 5 µM TRX for 30 min (PFK) or with 2.5 µM TRX for 20 min (MDH) at room temperature in a reaction buffer containing 0.1 M HEPES-KOH, pH 7.9 and 1 mM DTT. As control the extract was preincubated with 1 mM and 10 mM DTT without TRX. Recombinant Arabidopsis TRX isoforms TRX-m1, TRX-m4, TRX-x and TRX-f1 were produced in *E. coli* [18].

The preincubation reaction was mixed with reaction buffer and substrates to obtain a final concentration of 0.1 M HEPES-KOH, pH 7.9, 2 mM MgCl_2_, 0.15 mM NADH, 7.5 mM Fru-6-P, 1 U aldolase, 1 U triosephosphate isomerase and 1 U glycerol-3-phosphate dehydrogenase, and 2.5 mM ATP for PFK and 0.1 M HEPES-KOH, pH 7.9, 10 mM MgCl_2_, 0.2 mM NADPH and 1 mM oxaloacetate for MDH. Enzyme activity was spectrophotometrically determined at 340 nm (PFK) or 334 nm (MDH).

### 2.8. Sequence Alignments and Statistics

PFK protein sequences from *A. thaliana* (Prefix At) and *Oryza sativa* (Prefix Os) were obtained from public databases (www.arabidopsis.org, accessed on 21 February 2021; rice.plantbiology.msu.edu, accessed on 21 February 2021). Putative phosphofructokinase sequences from *Solanum lycopersicum* (Prefix Solyc), *Brachypodium distachyon* (Prefix Bradi), *Medicago truncatula* (Prefix Medtr), *Sorghum bicolor* (Prefix Sobic), *Chlamydomonas reinhardtii* (Prefix Cre) and *Volvox carteri* (Prefix Vocar) were obtained by BLAST search using the Phytozome10 database [41] with the *A. thaliana* AtPFK5 protein sequence as template. Sequence alignments of the protein sequences were made by using ClustalW [42]. The 3D-structural models of AtPFK3, AtPFK4 and AtPFK5 were predicted using Phyre2 [43], with default settings. The program used the crystal structure of the PFK from *Trypanosoma brucei* [44], the most similar protein among crystallized PFKs, for creating the model. Statistical analyses were made by use of the R program.

## 3. Results

### 3.1. Arabidopsis Contains Six Active PFK Isoforms

The Arabidopsis genome contains seven *PFK* genes, forming three distinct groups (Supplementary Figure S1 and [28,30]). We could previously demonstrate that all members of subgroup PFK_A, namely isoforms AtPFK1/3/4/6 and 7, have PFK activity through a plant expression system, namely transient transformation of tobacco leaves through Agrobacteria [28], while expression in *E. coli* did not result in active enzymes in our hands. Very recently, others were successful in expressing an active AtPFK5 in *E. coli* [32]. Additionally, the tagging of the enzyme at either the N-terminus or the C-terminus strongly inhibited its activity, it must therefore be expressed in its native, untagged form.

**Figure 1:**
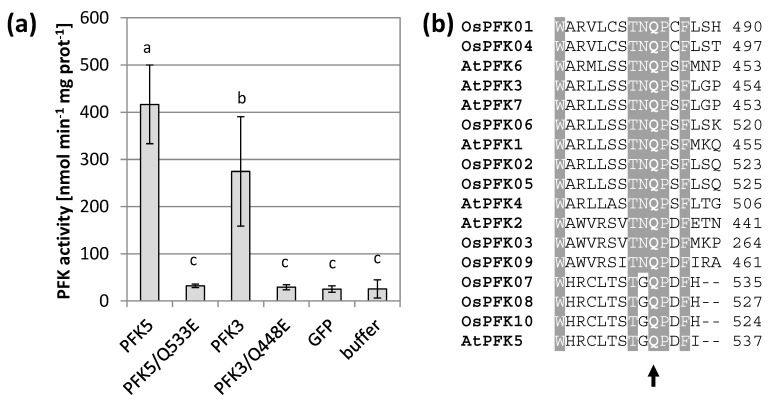
AtPFK5 is an active PFK. (**a**) PFK activity of tobacco leaves transiently transformed with *AtPFK5* and *AtPFK3* (WT form) compared with mutated variants (glutamine to glutamic acid at positions 533 and 448 for AtPFK5 and AtPFK3, respectively). The results are means ± SD. Different letters indicate significant differences at *p* < 0.05 (ANOVA, Post hoc Tukey test, *n* = 4). (**b**) Detail of the protein alignment with the conserved glutamine (Q) marked with an arrow for PFKs from *A. thaliana* (At) and *Oryza sativa* (Os).

In preparation for this work, we discovered that the reverse primer for *AtPFK5* differed from the sequence of the current genome version, thus causing a point mutation from glutamine to glutamic acid. After re-cloning and thus correction of the protein sequence, AtPFK5 showed high activity in transiently transformed tobacco leaves, at similar levels to AtPFK3 (Figure 1a). Since this residue is highly conserved among PFK enzymes (Figure 1b), we also mutated this residue in AtPFK3, which also completely inactivated the enzyme (Figure 1a). It can be therefore concluded that the conserved glutamine is a functionally essential amino acid in this enzyme family. In conclusion, Arabidopsis has not five, but six active PFK isoforms, among them two plastidic enzymes. It remains open whether AtPFK2 is an inactive enzyme or whether it lacks activity under the assay conditions.

### 3.2. Arabidopsis PFKs Are Sensitive to Redox Modifications

In order to explore whether PFK isoforms might respond to changes in their thiol redox status, we treated each isoform with redox-modifying chemicals after their expression in tobacco leaves. We also included the presumably inactive AtPFK2. Importantly, since we could not control for equal protein expression due to the absence of a tag, we divided each extract into several aliquots to determine differences due to the redox treatment. Additionally, we performed several rounds of independent transformations and summarized the results. During the extraction, the presence of low amounts of reducing agents, here 5 mM β-mercaptoethanol, were absolutely required to maintain PFK activity. Omission of this chemical lead to irreversible inhibition of the enzyme (data not shown).

**Figure 2:**
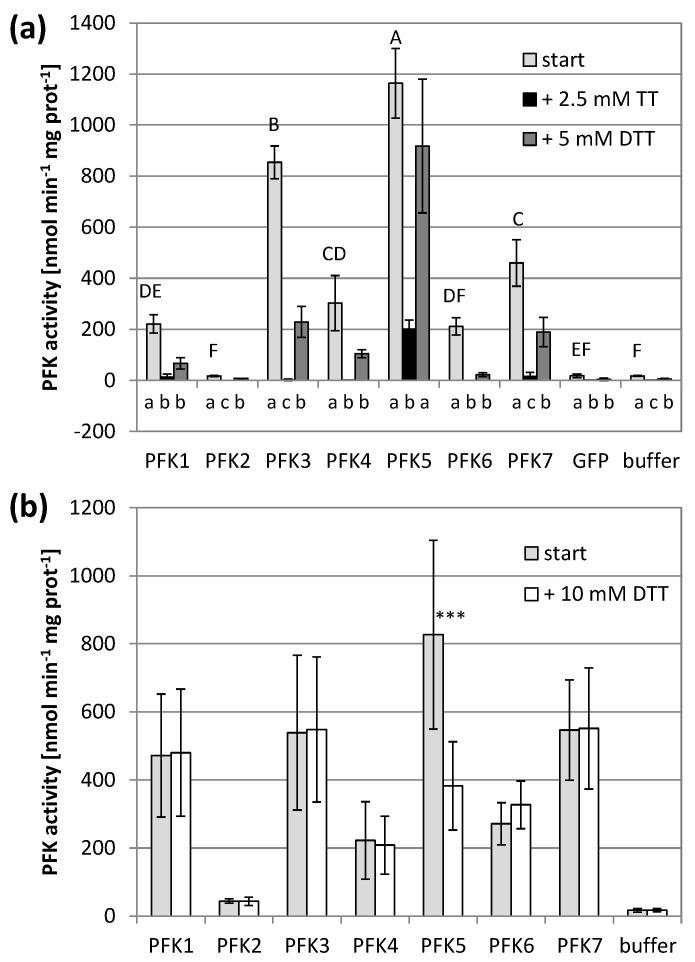
Redox sensitivity of *Arabidopsis thaliana* PFKs. (**a**) *A. thaliana* PFKs were transiently overexpressed in tobacco leaves. Leaves were extracted with buffer containing 5 mM β-mercaptoethanol (β-ME). After determining the initial activity, samples were oxidized with 2.5 mM sodium tetrathionate (TT) for 20 min and afterwards re-reduced with 5 mM dithiothreitol (DTT). The results are means ± SD of *n* = 3. Upper case letters show significant differences for each isoform to buffer and GFP control (*p* < 0.05, ANOVA and Post hoc Tukey test), lower case letters indicate significant differences among treatments for each protein isoform at *p* < 0.05 (ANOVA, Post hoc Tukey test). (**b**) *A. thaliana PFK1-7* were transiently overexpressed in tobacco leaves and extracted with 5 mM β-ME and additionally reduced under partially denaturing conditions with 10 mM DTT, 175 mM Bis-Tris propane (pH 9.0) and 500 mM NaCl. The results are means ± SD of *n* = 10. The asterisks (***) indicate a significant difference to untreated control at *p* < 0.05 (*t*-Test).

Application of the oxidizing agent sodium tetrathionate significantly decreased the PFK activity of all active PFKs. The five PFKs of the subgroup PFK_A completely lost their activity and were no longer significantly different from untransformed controls (Figure 2a). AtPFK2 remained inactive, and the AtPFK5 activity decreased, but still maintained some activity above the background (Figure 2a). Re-reducing the enzymes by 5 mM of the reducing agent dithiothreitol (DTT) restored PFK activity again, either partially for AtPFK3 and AtPFK7, or completely for AtPFK5, while AtPFK1, AtPFK4 and AtPFK6 were no longer active (Figure 2a).

The activity assay was modified in order to study the effect of reducing power. A previous study on NADP-MDH showed that monothiols such as β-mercaptoethanol were poorly capable of reducing the target enzyme even under high ionic strength [45]. We therefore treated the PFKs with 10 mM DTT under partially denaturing conditions, namely at pH of 9.0 in 500 mM NaCl (modified according to Scheibe et al. [39]). This treatment did not modify the activity of six PFKs, while it caused a significant activity decrease of the plastid AtPFK5, the only Arabidopsis member of the subgroup PFK_C (Figure 2b, Supplementary Figure S1).

### 3.3. PFK Isoforms Contain Multiple Conserved Cys

Since we observed modifications in enzyme activities among PFK isoforms by redox-active substances, the occurrence of Cys as potential targets for thiol-mediated redox regulations was analyzed in Arabidopsis and rice PFKs, as well as in other plant species including the two algae *Chlamydomonas reinhardtii* and *Volvox carteri*. Indeed, several Cys are present in different PFK isoforms, with varying degree of conservation. A CGGL/IC motif was conserved among all PFKs, including the related outgroup PFK from *Trypanosoma brucei* (Supplementary Figure S3; Cys187 and Cys191 in AtPFK5). This motif was accompanied by the presence of a partially conserved vicinal Cys. Another strongly conserved Cys was present towards the C-terminal end of the proteins (Supplementary Figure S3; Cys373 in AtPFK5), sometimes accompanied by a second adjacent Cys. A third highly conserved Cys locates close to the C-terminus (Supplementary Figure S3; Cys475 in AtPFK5). In subgroup PFK_C including the plastidic AtPFK5, a second motif with two Cys was discovered, CXDXXC (Supplementary Figure S3; Cys152 and Cys157 in AtPFK5).

**Figure 3:**
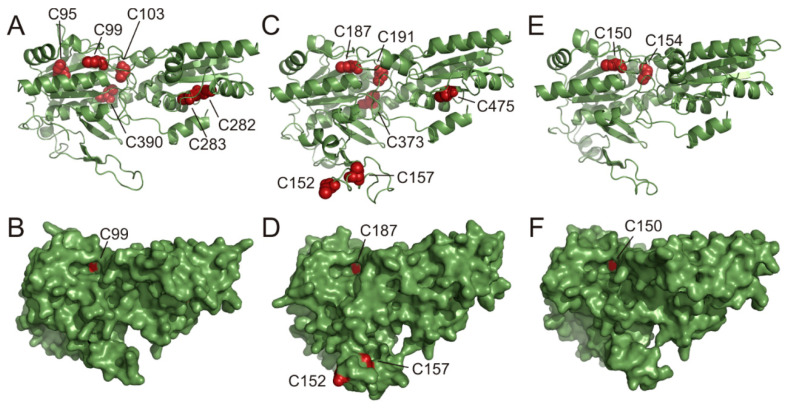
Predicted AtPFK3, AtPFK4 and AtPFK5 3D structures. Based on the 42% identity to the amino acid sequence of the PFK apoenzyme from *Trypanosoma brucei*, the AtPFK3 (**A**,**B**), AtPFK5 (**C**,**D**) and PFK4 (**E**,**F**) structures were predicted using Phyre2 software. (**A**,**C**,**E**) Ribbon representation of the protein architecture, with conserved Cys and their position in red. (**B**,**D**,**F**) Solvent-excluded surface with the Cys C152 and C157 in red, representing the AtPFK5 CXDXXC motif.

Based on the amino acid sequences of AtPFK3, AtPFK4 and AtPFK5, the 3D-structures of these proteins were predicted using Phyre2 [43]. The three-dimensional structure was based on a 42 % identity with the PFK apoenzyme from *T. brucei* [44,46]. The surface structure of the protein revealed that out of several conserved Cys only two Cys at the positions 152 and 157 from the AtPFK5 CXDXXC motif were located in an unstructured and flexible loop (Figure 3C,D). The predicted surface display of the CXDXXC motif suggests that these Cys are easily accessible for redox-regulating proteins such as TRXs. In contrast, other conserved Cys were predicted to be buried in the protein, and only AtPFK3-Cys99 and AtPFK5-Cys187 of the CGG(L/I)C motif were visible in a protein cavity of the proposed active center of the enzyme.

### 3.4. Mutations of Conserved Cys Modify Enzyme Activity

In order to address Cys with possible importance for redox sensitivity of AtPFKs, selected residues were mutated to serine in AtPFK3 (cytosolic isoform) and AtPFK5 (plastidic isoform) to mimic a constitutively reduced state. The constructs were transiently transfected into tobacco leaves, and enzyme activities determined in extracts with different redox-active chemicals.

**Figure 4:**
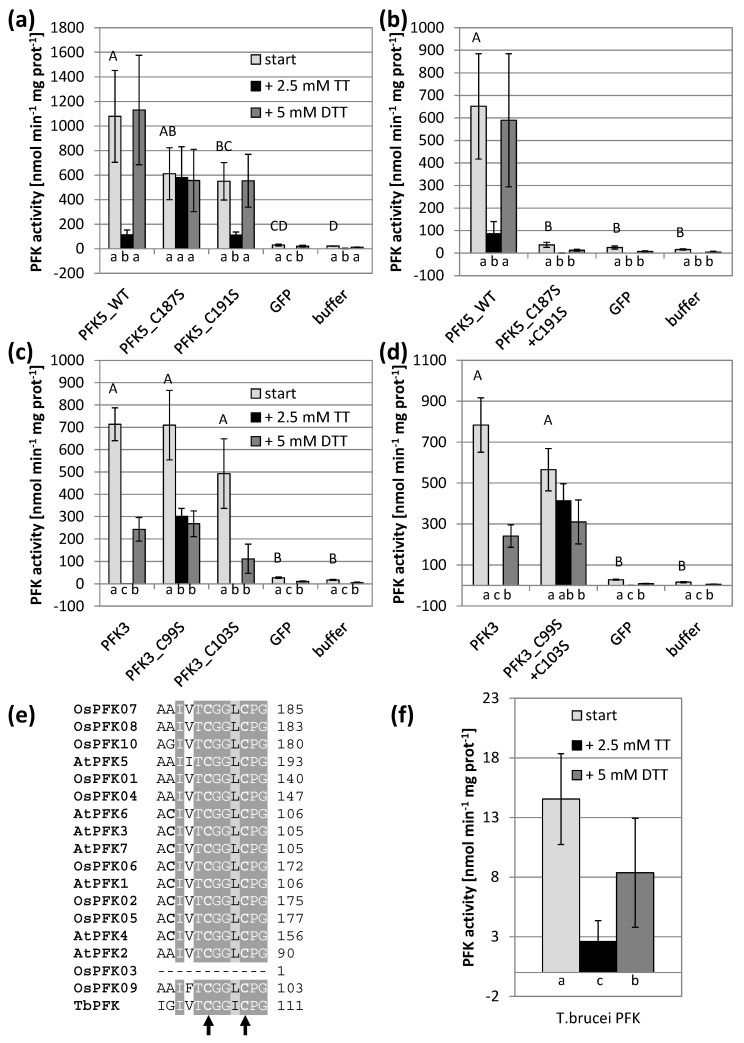
Impact of the CGG(L/I)C motif on PFK activity and oxidation sensitivity. Cys to serine single and double mutations were introduced in the CGG(L/I)C motif of *A. thaliana* AtPFK3 and AtPFK5 proteins. The variants were transiently overexpressed in tobacco leaves (**a**–**d**). Wildtype PFK from *T. brucei* (**f**) as well as PFKs from the tobacco leaf samples (**a**–**d**) were extracted with buffer containing 5 mM β-mercaptoethanol (β-ME). After initial activity determination, samples were oxidized with 2.5 mM sodium tetrathionate (TT) for 20 min, and afterwards re-reduced with 5 mM dithiothreitol (DTT). Upper case letters indicate significant differences for each isoform to buffer and GFP control, lower case letters indicate significant differences among treatments for each isoform at *p* < 0.05 (ANOVA, Post hoc Tukey test, *n* = 3–6). The results are means ± SD. (**e**) Detail of the protein alignment of the CGG(L/I)C motif in PFKs from *A. thaliana*, *O. sativa* and *T. brucei*. The two mutated Cys are marked with an arrow.

First, the highly conserved CGG(L/I)C motif was targeted through mutations. Interestingly, mutation of the first Cys of the motif in both isoforms resulted in reduced sensitivity against oxidation in both AtPFK3 and AtPFK5, while this was not the case when serine substituted the second Cys (Figure 4a,c). When both Cys were mutated to serine, the enzyme activity of AtPFK5 drastically decreased, while AtPFK3 remained active and again oxidation-insensitive (Figure 4b,d). Since this motif was also present in the protist *T. brucei* (Figure 4e), whose PFK sequence is similar to plant PFKs, we tested whether TbPFK was also sensitive to oxidation. Indeed, oxidation with 2.5 mM sodium tetrathionate significantly inhibited PFK activity in *T. brucei* extracts, which was partially restored by re-reduction with 5 mM DTT (Figure 4f).

In addition, other conserved Cys were mutated to serine and activity was analyzed. However, neither modification of Cys373 nor of Cys475 in AtPFK5 significantly modified enzyme activities under normal or oxidizing conditions (Supplementary Figure S4A). In AtPFK3, mutation of Cys390 or C95 had no effect (Supplementary Figure S4B), but the mutation of Cys282/Cys283 corresponding to Cys373 in AtPFK5 strongly decreased protein activity (Supplementary Figure S4C).

**Figure 5:**
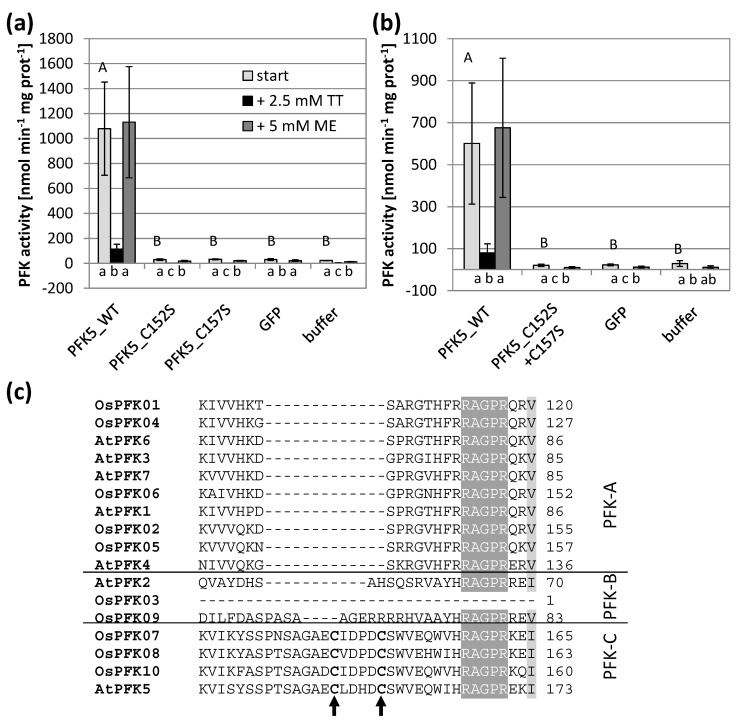
Impact of the CXDXXC motif on PFK activity and oxidation sensitivity. Single (**a**) and double (**b**) Cys to serine mutations were introduced in *A. thaliana* AtPFK5 proteins with CXDXXC motif. The variants were transiently overexpressed in tobacco leaves. Extraction buffer contained 5 mM β-mercaptoethanol (β-ME). After determining the initial activity, samples were oxidized with 2.5 mM sodium tetrathionate (TT) for 20 min and afterwards re-reduced with 5 mM dithiothreitol (DTT). Upper case letters show significant differences for each isoform to buffer control, lower case letters indicate significant differences among treatments for each protein at *p* < 0.05 (ANOVA, Post hoc Tukey test, *n* = 3). (**c**) Detail of the protein alignment of the CXDXXC motif in PFKs from *A. thaliana* and *O. sativa*. The two mutated Cys are marked with an arrow.

Since only AtPFK5 displayed sensitivity towards thiol reduction (Figure 2b), we were especially interested in the CXDXXC motif which was specific for this subgroup. Indeed, changing either of the two Cys or both into serine in order to mimic a constitutively reduced state resulted in complete loss of activity which was no longer different in comparison to untransformed controls (Figure 5). This observation suggests that reduction of the enzyme at that motif deactivates the enzyme, as it has been described for enzymes of the oxidative pentose phosphate pathway.

**Figure 6:**
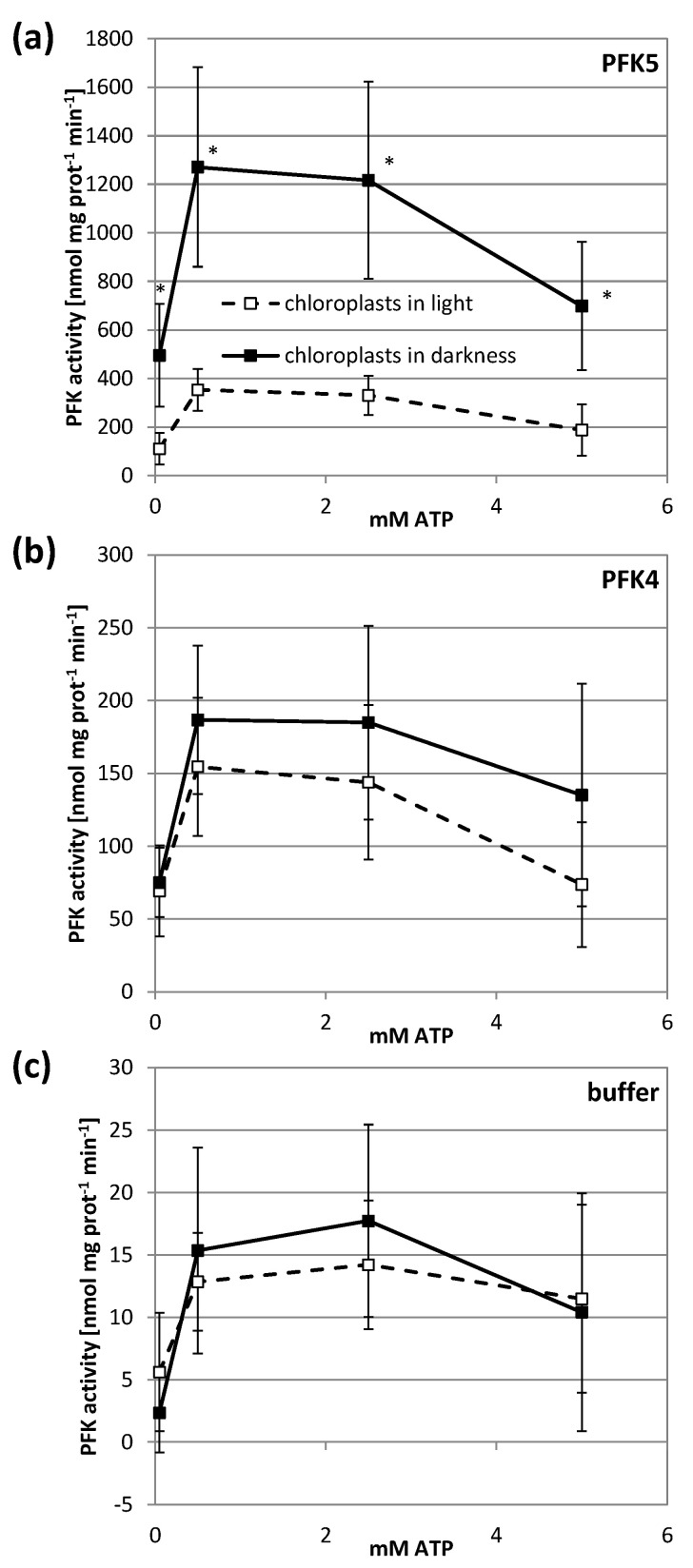
Light inactivation of AtPFK5 in tobacco chloroplasts. *AtPFK5* (**a**) and *AtPFK4* (**b**) were transiently overexpressed in tobacco leaves. Leaves expressing AtPFK4 as well as plants infiltrated with the infiltration buffer (**c**) were used as negative controls. Chloroplasts were isolated and illuminated with 1000 µmol photons m^−2^ s^−1^ for 1 h (□) or kept in darkness (■). PFK activity was measured under various ATP concentrations. The results are means ± SD of *n* = 10. Significant differences between light and darkness treatment at each ATP concentration are marked with asterisks (*) (*t*-Test, *p* < 0.001).

Inhibition of a plastid enzyme under reducing conditions might indicate an inhibition under physiological conditions in the light. We therefore used chloroplast suspensions from transiently transformed tobacco leaves that either expressed AtPFK5 or the other plastidic isoform AtPFK4, and kept them in either darkness or light for 1 h. In this experiment, the activity assay was performed with several ATP concentrations in order to exclude affinity differences for this substrate. Illumination of chloroplasts strongly inhibited AtPFK5 activity at either ATP concentration, while it had no significant effect on AtPFK4 or the native tobacco chloroplast PFK activity (Figure 6).

### 3.5. AtPFK5 Is a Target of Thioredoxin-f1

The experiments described above suggested a redox-dependent modification of AtPFK5 activity. We therefore analyzed whether specific TRXs might be involved in this modification. Four different TRXs were tested, namely TRX-f1, TRX-x, TRX-m1 and TRX-m4. As a positive control, NADP-MDH activity whose TRX-dependent activation is well-described [12], was measured in parallel, in order to confirm correct treatment conditions. PFK activities of AtPFK4- and AtPFK5-expressing tobacco leaves were analyzed as before and compared with untransformed controls.

**Figure 7:**
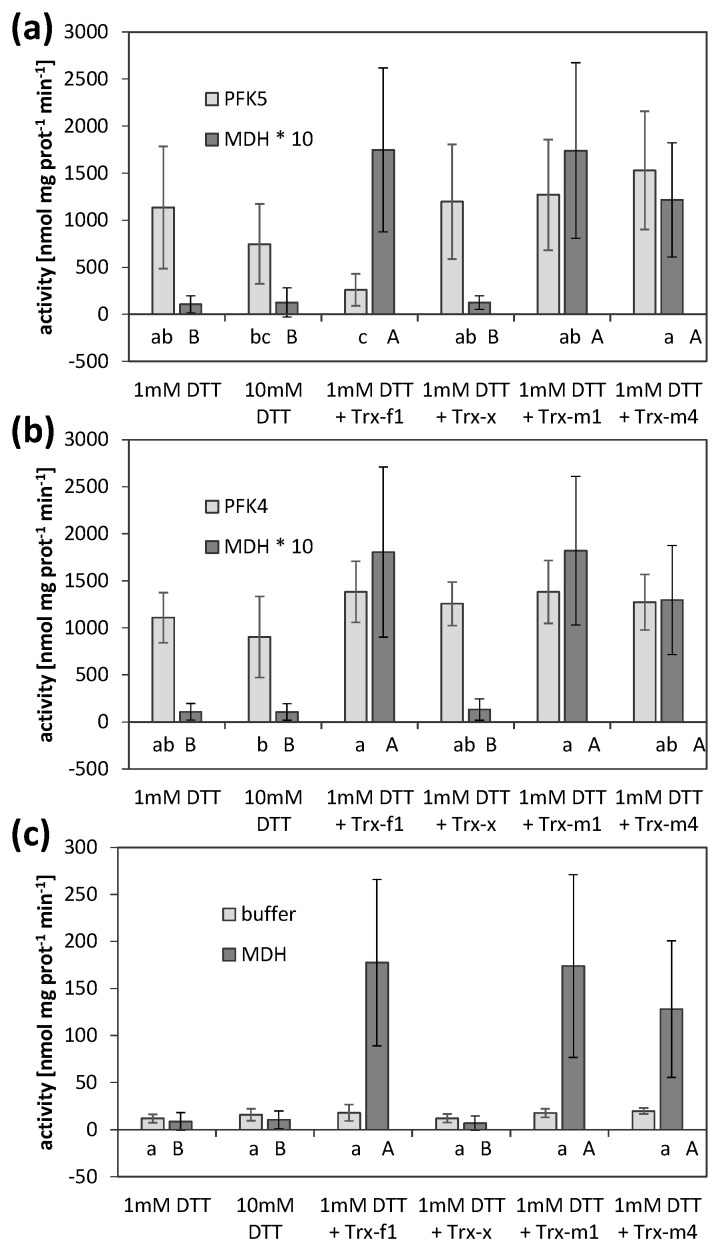
Thioredoxin-mediated inactivation of AtPFK5. Activities of PFK and NADP-MDH in extracts of tobacco leaves transiently overexpressing either AtPFK5 (**a**) or AtPFK4 (**b**), or mock controls (**c**). Crude leaf extracts were incubated with 1 and 10 mM DTT, or with 1 mM DTT and 5 µM (PFK) or 2.5 µM (MDH) of the respective TRX, and activity was measured after 30 min of incubation. Results are means ± SD of three independent infiltrations with three samples each. Upper case letters indicate significant differences among treatments for MDH, lower case letters indicate significant differences among treatments for PFK at *p* < 0.05 (ANOVA, Post hoc Tukey test). For comparability, MDH activity was multiplied by 10 (*) to be in the same range as PFK activity in (**a**,**b**).

AtPFK5 activity was high at the beginning of the treatment under reducing conditions (1 mM DTT) and NADP-MDH was inactive (Figure 7). Incubation of the extracts with TRX-f1 significantly inhibited AtPFK5 activity while it activated NADP-MDH (Figure 7a). However, TRX-m1 and TRX-m4 also activated NADP-MDH, while those TRXs did not influence AtPFK5. TRX-x did not modify any of the enzymes tested. AtPFK4 or native tobacco activities remained unchanged in the presence of any of the TRXs, although NADP-MDH in the same incubations responded as expected (Figure 7b,c).

### 3.6. A Knockout of AtPFK5 Has Little Effects in Arabidopsis Plants

**Figure 8:**
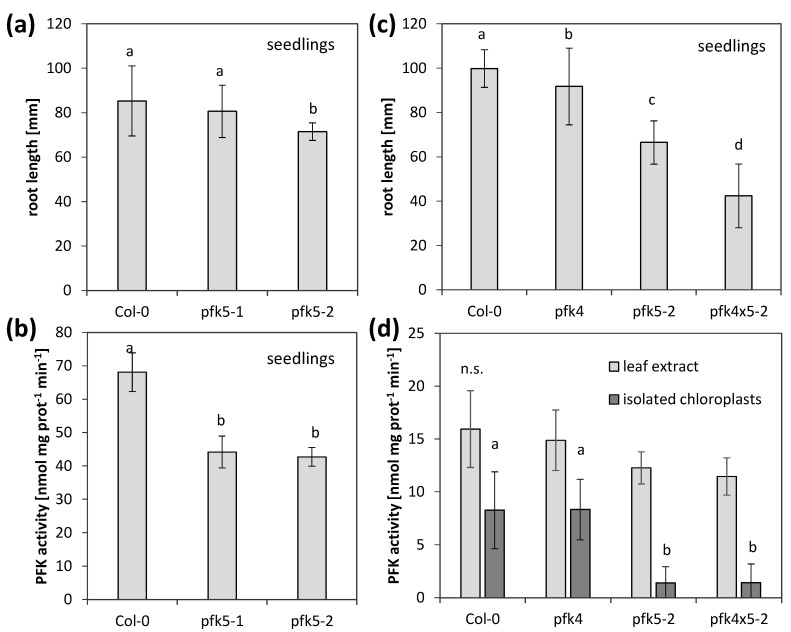
Analysis of two independent *PFK5* T-DNA insertion mutants. (**a**) *A. thaliana* seedlings of *pfk5-1* (Salk_067384) and *pfk5-2* (Sail_694_A07) were grown under long-day conditions on solid 1/10 Hoagland medium together with the wildtype Col-0. After 14 days, root length was determined. Data represent means ± SD of 30 plants. Letters indicate significant differences at *p* < 0.05 (ANOVA, Post hoc Tukey test). (**b**) PFK activity of 14-day-old seedlings of Col-0, *pfk5-1* and *pfk5-2* grown on 1/10 Hoagland medium. Different letters indicate significant differences in protein activity at *p* < 0.05 (ANOVA, Post hoc Tukey test, *n* = 3–4). (**c**) Root length of 14-day-old seedlings, grown under long-day conditions on solid 1/10 Hoagland medium. Col-0 wildtype was compared to *pfk5-2* (Sail_694_A07), *pfk4-1* (SALK_012602) and the double mutant *pfk5-2 pfk4-1*. Data represent means ± SD of 3 independent experiments with 20 individuals each. Letters indicate significant differences at *p* < 0.05 (ANOVA, Post hoc Tukey test). (**d**) PFK activity in crude leaf extracts and isolated chloroplasts from 5-week-old rosette leaves of *pfk4-1*, *pfk5-2* and the double mutant in comparison to wildtype Col-0. Data are means ± SD. Letters indicate significant differences among genotypes at *p* < 0.05 (ANOVA, Post hoc Tukey test, *n* = 4–6). n.s., not significant.

Since AtPFK5 is the only member of the subgroup PFK_C in Arabidopsis and the only redox-regulated PFK, we were interested to see whether its activity is important for plant growth and metabolism. We therefore isolated two T-DNA knockout mutants for *AtPFK5*, *pfk5-1* (SALK_067384) and *pfk5-2* (SAIL_694_A07) (Supplementary Figure S2A), and confirmed their homozygous status by genotyping. The transcript amount of *AtPFK5* was strongly decreased in *pfk5-1*, while it was no longer detectable in *pfk5-2* (Supplementary Figure S2B). The overall activity of PFK in 7-day-old seedlings was slightly, but significantly decreased in both genotypes (Figure 8b), indicating that AtPFK5 contributes to overall PFK activity. Only in *pfk5-2*, the root length on Hoagland medium was significantly reduced, in comparison to the wildtype (Figure 8a).

In order to determine the relative importance of AtPFK5 for overall plastid PFK activity, we additionally isolated a mutant in the other plastidic PFK, *AtPFK4* (Supplementary Figure S2C) and confirmed it as a knockout (Supplementary Figure S2D). Subsequently, the double mutant *pfk5-2 pfk4-1* was created by crossing. In this double mutant, root length of seedlings was strongly reduced (Figure 8c), while overall PFK activity was not further reduced (Figure 8d). In these plants, PFK activity was also measured in isolated chloroplasts, and a significantly lower activity was only observed for *pfk5-2* or the double mutant *pfk5-2 pfk4-1* (Figure 8d), suggesting that AtPFK5 mainly contributes to plastid PFK activity.

**Figure 9:**
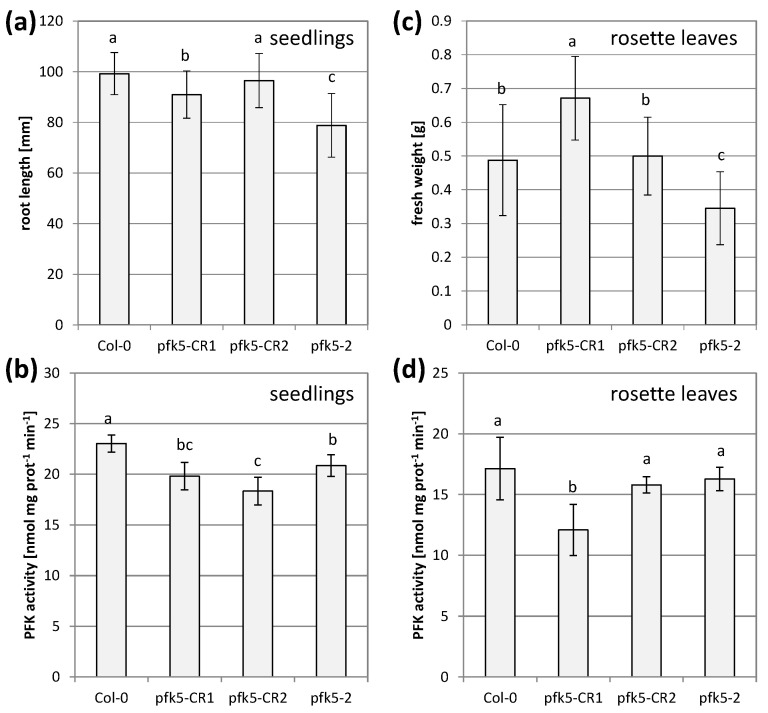
Analysis of two independent *PFK5* CRISPR lines. (**a**) *A. thaliana* seedlings of *pfk5-CR1* and *pfk5-CR2* were grown under long-day conditions on solid 1/10 Hoagland medium together with *pfk5-2* and the wildtype Col-0. After 14 days, root length was determined. Data represent means ± SD of 3 independent experiments with 20 individuals each. Letters indicate significant differences at *p* < 0.05 (ANOVA, Post hoc Tukey test). (**b**) PFK activity of 7-day-old seedlings of the four genotypes grown on MS medium. Different letters indicate significant differences in protein activity at *p* < 0.05 (ANOVA, Post hoc Tukey test, *n* = 5–6). (**c**) Rosette weight of 5-week-old plants of the four genotypes grown on soil. Data are means ± SD of 12 plants. Letters indicate significant differences at *p* < 0.05 (ANOVA, Post hoc Tukey test). Images of the plants are shown in Supplementary Figure S5. (**d**) PFK activity in crude leaf extracts from 5-week-old rosette leaves of the four genotypes. Data are means ± SD. Letters indicate significant differences among genotypes at *p* < 0.05 (ANOVA, Post hoc Tukey test, *n* = 4).

During these experiments, there was always a discrepancy between the level of PFK activity and growth. Severe growth reductions in the double mutant did not correlate with remaining PFK activity. The mutant line *pfk5-2* most likely had another mutation in the gene AT2G45330 (www.arabidopsis.org, accessed on 23 February 2021), coding for a tRNA-2′-phosphotransferase, which was described as embryo-lethal [47]. This insertion was putatively linked with the *AtPFK5* locus and was not removable by crossing. Therefore, we additionally created two independent CRISPR mutant lines. A point mutation in the third exon of the protein (*pfk5-CR1*) and a point mutation in the second last exon (*pfk5-CR2*) were successfully isolated. All three mutants, *pfk5-2* as well as both CRISPR lines exhibited slightly, but significantly reduced PFK activities (Figure 9b), but the CRISPR lines were much less affected in root growth than *pfk5-2* (Figure 9a).

In rosette leaves of five-week-old plants, overall PFK activity was even less affected, and only *pfk5-CR1* showed significant activity reduction (Figure 9d), while *pfk5-CR2* and *pfk5-2* were not affected in rosette leaf PFK activity (Figure 8d, Figure 9d). Despite this observation, *pfk5-2* still showed a growth defect (Figure 9c, Supplementary Figure S5). These results suggest that background mutations might cause the phenotype in *pfk5-2*, and not the decrease in AtPFK5 expression or activity.

The observation that knockout of *AtPFK5* caused only a reduction in seedling PFK activity, but hardly in rosette leaf activity, let us to check its tissue specific expression by available expression data. A cell-type specific dataset of both roots and shoots revealed higher expression of *AtPFK5* in roots than in leaves (Supplementary Figure S6), hinting at a low expression level in leaves. Interestingly, there was tissue-specific expression of *AtPFK5* in leaves, namely in phloem-associated cells, suggesting a role in specific transport or phloem loading processes. In order to get a first hint on this phenomenon, the sugar content in rosette leaves of the different mutant types was analyzed at the end of the 8-h-illumination period, before the lights went off. Surprisingly, glucose accumulated significantly in both CRISPR lines, while other sugars and starch content were not affected (Supplementary Figure S7). The lower-growing line *pfk5-2*, on the other hand, showed a tendency for lower carbohydrate levels, but those differences were not significant.

## 4. Discussion

### 4.1. Redox Sensitivity of Plant and Trypanosoma PFKs

The activity of several enzymes of plant primary metabolism responds to the cellular or subcellular redox environment. In this work, we were interested to study the redox sensitivity of plant PFKs. Redox sensitivity was reported in early works on plant PFKs [25,26,27] and was also described for rabbit muscle PFK [48]. However, these described effects were dissimilar. While rabbit muscle PFK was active when fully reduced, pea and spinach leaf PFK from chloroplasts was inactivated by reducing conditions [25,26,27]. Our results demonstrate that both phenomena are actually valid and can be traced back to specific parts of the protein.

Cys residues belong to those amino acid residues represented least in proteins indicating specific functions in structure formation, activity or regulation [3]. Interestingly, during evolution Cys content in proteins increased [49]. Cys participating in disulfide bridges are more likely to be conserved compared to non-regulatory Cys [50,51]. Sequence analysis of monocotyledonous, dicotyledonous and algae PFKs revealed several conserved Cys (Supplementary Figure S3). Besides positionally conserved single Cys the study identified two distinct Cys-containing motifs. The CGG(L/I)C motif is almost ubiquitous in all plant PFKs, and additionally in the PFK from the parasite *T. brucei*, whereas the CXDXXC motif is strongly conserved in the plastid-localized members of the subgroup PFK_C from higher plants (Supplementary Figure S1).

At the start of our experiments, PFK activity was quantified under oxidizing and reducing conditions. The activity of six *A. thaliana* PFKs was dependent on extraction under reducing conditions, here achieved with β-mercaptoethanol, which might protect the cysteines of the CGG(L/I)C motif against mono-Cys oxidation. The activity of all PFKs was considerably inhibited by subsequent oxidation with sodium tetrathionate (Figure 2a), similar to observations made for rabbit muscle PFK [48]. Only AtPFK5 maintained some activity when oxidized with sodium tetrathionate. With the subsequent re-reduction by DTT, enzymatic activity could partially be restored for AtPFK3 and AtPFK7, or completely in the case of AtPFK5 (Figure 2a). However, as described below, this oxidation sensitivity might not be a regulatory mechanism, but rather an experimental artifact.

Site-directed mutagenesis is a classical method to identify thiol reagent-sensitive Cys and examine structural protein functionality. For this analysis, the plastid-localized AtPFK5 and cytosolic AtPFK3 were selected, and mutated variants were analyzed under slightly reducing and oxidizing conditions. Mutation of the first Cys, namely AtPFK5-Cys187 and AtPFK3-Cys99, of the CGG(L/I)C motif generated variants which showed significantly less activity inhibition by oxidation compared to the corresponding native protein (Figure 4a,c). Nevertheless, the mutation of the second closely located Cys did not affect protein activity (Figure 4a,c). Since this motif is most likely not accessible for TRXs due to its position inside the protein structure (Figure 3), and AtPFK4 with this motif is not modified in activity upon TRX treatment (Figure 7), it is suggested that the Cys in the CGG(L/I)C motif are no target of a controlled redox regulation. The activity loss upon oxidization is probably due to the location of that Cys close to the active center of the PFK protein, namely Cys187 of AtPFK5 is very close to the ATP binding site Gly189 [28]. It remains unclear why the activity of AtPFK5 is less sensitive to oxidation than the other AtPFK isoforms (Figure 2). Potentially, the slightly modified amino acid composition close to this region (Ile185 in AtPFK5, but Val in all others) could be involved in this phenomenon. Therefore, this region might be responsible for oxidation sensitivity of PFKs in plants as well as in *T. brucei* (Figure 4f).

Similar effects have been reported for many other proteins where a change of the activity was linked to modification of a Cys thiol (e.g., [3,52,53,54]). In the case of NADP-dependent malate dehydrogenase, the iodoacetamide-based alkylation of the Cys175 located in the active center prevented the conformational change of the catalytic complex [55]. The sodium tetrathionate-linked sulfenylation of the Cys [56] likely impaired the ATP-binding in the active center of the proteins. In the PFKs with known protein structure from *E. coli* and *T. brucei*, the second glycine of the CGG(L/I)C motif is associated with the ATP-binding of the PFK protein and the neighboring Cys might be involved in ATP-binding [44,46,57].

In contrast, due to the lacking Cys, the AtPFK5-C187S and AtPFK3-C99S mutants are insensitive to the sulfenylation, and the PFK activity is maintained upon oxidation. This is also true for the double mutant AtPFK3-C99S-C103S. However, the AtPFK5-C187S-C191S double mutant was no longer active, which could be due to drastic mutation-linked conformational changes of the proteins resulting in the loss of protein functionality (Figure 4b). We therefore conclude that the CGG(L/I)C motif is not a site of redox-regulation by the FDX-TRX system, but the reduced state of the first Cys is important for enzyme activity, also indicated by the high conservation across isoenzymes and species (Supplementary Figure S3).

### 4.2. Plastidic PFKs Are Redox Regulated through the CXDXXC Motif

Plastid-localized AtPFK5 was the only isoform that was inhibited after exposure to reducing conditions, namely by 53% (Figure 2b). Similar results were achieved for G6PDH, where 25 to 35 % of enzyme activity was lost upon reduction with DTT in crude leaf extracts [11]. The observed inhibition by DTT raised the possibility that AtPFK5 could be deactivated in chloroplasts under illumination, thus mimicking a behavior similar to enzymes of the oxidative pentose phosphate pathway. Indeed, members of subgroup PFK_C contain a region in their protein sequence, the CXDXXC motif, which is not present in cytosolic PFKs and could represent an insertion that occurred after amplification of the gene family. This insertion has a different sequence in algal PFKs and is absent in PFK from *T. brucei* (Supplementary Figure S3), but it is strongly conserved in plastid-localized PFKs of independent subgroup PFK_C members (Supplementary Figure S3). A similar insertion event of a redox regulatory side has been suggested for plastid-localized FBPase [13]. Homology modelling of this region predicts that the Cys residues are exposed at the protein surface and could be well accessible to TRXs (Figure 3).

Calculation revealed a distance of 8.9 Å between the two Cys residues AtPFK5-Cys152 and AtPFK5-Cys157 in the CXDXXC motif. Since the distance between two thiols forming a disulfide typically falls within 4.4 and 6.8 Å in 95 % of all investigated refined proteins, formation of a spontaneous disulfide bridge is unlikely [58], but a catalyzed disulfide bridge might still be possible. Indeed, binding of TRX to CF1 γ-subunit of ATPase induces conformation changes of the γ-subunit [59,60], suggesting a similar structural change in potential TRX-linked regulation of AtPFK5 that could lead to a lower distance upon redox modification. In G6PDH, redox regulation is facilitated through a C(X)_7_C-disulfide bridge in *Solanum tuberosum* and *A. thaliana* [61,62], which is an even wider distance compared with AtPFK5. Furthermore, prediction of the AtPFK5 protein structure could be erroneous due to low conservation of this loop in comparison to the crystallized *T. brucei* PFK that was used as the basis for prediction. In addition, intermolecular disulfide bridges cannot be excluded, since PFKs often act as oligomers. For example, *T. brucei* PFK forms tetramers [44]. A crystal structure or extensive protein modeling of AtPFK5 would be required to solve these open questions and to determine the influence of this redox regulation on protein structure and activity.

Further experiments supported the hypothesis that the CXDXXC motif might be responsible for redox regulation of AtPFK5. Single and double mutations of the Cys into serine, namely in the variants AtPFK5-C152S, AtPFK5-C157S and AtPFK5-C152S-C157S, resulted in inhibition of AtPFK5 activity under all assay conditions (Figure 5a,b). This suggests that maintaining the conformation of the reduced state and preventing the formation of a disulfide bridge through our mutational approach strongly impacts AtPFK5 activity. In comparison, double mutation of regulatory Cys C149S/C157S of light-inactivated *A. thaliana* G6PDH resulted in almost complete loss of activity and insensitivity towards redox regulation [63]. It would be interesting to investigate a conformation mimicking the constitutively oxidized form, e.g., by introducing a salt bridge between a positive and negative amino acid residue substituting for both Cys residues and, therefore, active state of PFK since this approach could add another proof for our hypothesis. A similar approach was used for maintaining a pseudo-oxidized conformation in 2-Cys peroxiredoxin in the C54D/C176K double mutant [64].

Initially it had been observed that PFK activity from crude leaf extracts could be completely inactivated in light and upon reduction with DTT [25]. Further experiments demonstrated that this inhibition is especially observed in PFK isolated from chloroplasts [27]. For chloroplasts isolated from *Spinacia oleracea*, a treatment with physiologically relevant low concentrations of NADPH strongly inhibited the protein, tentatively indicating that NADPH-dependent TRX reductase and TRXs are involved in redox regulation of the PFK [26]. The NADPH-inactivated PFK exhibited a higher K_m_ value for its substrates ATP and Fru-6-P [26]. In our study, chloroplasts transiently overexpressing AtPFK5 showed a significant inhibition of PFK activity by 66-78% upon illumination, but without a strong influence of the ATP concentration in the assay (Figure 6a). AtPFK4 was selected as a negative control, because it is plastid-localized but does not have the CXDXXC motif. For the AtPFK4-expressing tobacco leaves, no significant inhibition of PFK activity could be detected after light treatment of chloroplast (Figure 6b,c).

As further evidence, an influence of different TRXs on AtPFK5 was analyzed. While 1 mM DTT alone did not inhibit AtPFK5 activity, the co-incubation of TRX-f1 with AtPFK5 and 1 mM DTT resulted in significant inactivation of the enzyme, which was not observed for AtPFK4 (Figure 7). TRX-m1 and TRX-m4 did not affect AtPFK5 activity, while they activated NADP-MDH, the positive control in our assay in order to proof activity of the TRXs used. NADP-MDH has previously been identified as regulated by TRX-f1, -f2, -m1, -m2 and -m4 (summarized in [2,6]). G6PDH is regulated by TRX-f and -m [62]. FBPase, however, the enzyme catalyzing the reverse reaction of AtPFK5, is most efficiently regulated by TRX-f1 and -f2, which is similar to the induction pattern in this study. None of the enzymes was activated by TRX-x.

The experiments described here clearly support our hypothesis that chloroplast AtPFK5 is redox-regulated by TRX-f, being deactivated under reducing conditions such as illumination. This hypothesis is intriguing since a degradation of hexoses through chloroplast glycolysis during the day in order to produce NADH is not beneficial for plant cells, and also the degradation of glucose through the oxidative pentose phosphate pathway is deactivated during the day through the TRX system. In accordance with our result, another group very recently provided independent evidence that AtPFK5 is regulated through TRX-f, using recombinant protein expressed in *E. coli*, and in this way strengthens our findings [32]. Another evidence for our hypothesis comes from a recent proteome study for interactors of 2-Cys peroxiredoxin, the protein involved in re-oxidation of plastid proteins [65]. In this study, AtPFK5 was found as an interactor of 2-Cys peroxiredoxin. Indeed, 2-Cys peroxiredoxin might be involved in formation of the disulfide bridge of AtPFK5 and thus might activate this enzyme in the dark. The functionality of this interaction was recently confirmed [32]. However, in earlier proteome surveys on chloroplast redox modifications through TRXs, AtPFK5 was not found. This could be due to the fact that the protein is expressed only in specific cells of a leaf, namely the phloem-associated cells (Supplementary Figure S6, [66,67]), and therefore might be only present at low levels.

### 4.3. What Could Be the Function of Redox-Regulated AtPFK5?

Our study complements previous results on G6PDH. Both, AtPFK5 and G6PDH, the two major hexose-degrading enzymes in the chloroplast, are now established targets of TRX-dependent redox regulation. These enzymes are inactivated by reducing conditions under illumination through the TRX system. G6PDH functions in the oxidative pentose phosphate pathway and sugar catabolism during the night [10,11,61,62,63,68]. The physiological function of the redox regulation of AtPFK5 needs to be discussed. Inactivation of AtPFK5 during the day with corresponding activation of the counterpart FBPase would facilitate starch production [69,70]. However, expression of the *AtPFK5* gene is lower in leaves than in non-green tissues (Supplementary Figure S6, [66]) suggesting a minor role in photosynthesizing organs. A role for AtPFK5 in sugar export during the night is at least in *A. thaliana* unlikely, because most of the sugar is exported as maltose during the night via the transporter MEX1 [71,72]. Taken into account that expression of *MEX1* but not *AtPFK5* in *A. thaliana* is diurnally regulated [73,74], AtPFK5 most likely has another function.

The high expression of *AtPFK5* in leaf phloem companion cells (Supplementary Figure S6, [67,75]) suggests a specific role in phloem loading or a phloem-related process. The co-expression with the isocitrate dehydrogenase genes *IDH1* (*At4g35260*), *IDH2* (*At2g17130*) and *IDH-VI* (At3g09810) (http://atted.jp/, accessed on 23 February 2021), which are also highly expressed in this cell type [67], might hint at a function in energy production in leaf phloem companion cells. However, mutants in *AtPFK5* show only mild phenotypes, i.e., slightly reduced root growth, and no effect on rosette leaf growth (Figure 9). Similarly, the knockout of plastid NADP-MDH did not modify plant growth or phenotype, indicating multiple compensatory mechanisms in plant primary metabolism [76].

However, the data also suggest that AtPFK5, and not AtPFK4, is the major plastid isoform, since the chloroplast PFK activity was similarly low in *pfk5-2* and the double mutant *pfk5-2 pfk4-1* (Figure 8d). A slight accumulation of glucose in rosette leaves of the CRISPR/Cas9 knock out lines (Supplementary Figure S7) hints at some irregularities in sugar metabolism and/or transport in *AtPFK5* mutants, but this requires further studies. In addition, higher-order mutants where all members of the subgroup PFK_A are knocked out would enable to study AtPFK5 in more detail at the whole-plant level.

## 5. Conclusions

Phosphofructokinases have long been suggested to be regulated by the redox status of the cellular compartment. In this study, we present evidence that oxidation sensitivity is due to a Cys close to the reaction center of the enzyme. However, this might not be a TRX-regulated process. On the other hand, the sensitivity of plastid PFKs to reducing conditions could be linked to the CXDXXC motif that is present in a flexible loop at the surface of the protein and is likely a target of TRX-f. This thiol switch mechanism enables plants to deactivate glycolytic glucose breakdown in chloroplasts via AtPFK5 in times of high availability of reducing power, such as in day time.

## Data Availability

Data is contained within the article or supplementary material.

## Supplementary Materials

The following are available online at https://www.mdpi.com/2076-3921/10/3/401/s1, Figure S1: Phylogeny of plant phosphofructokinase isoenzymes. Figure S2: Genotype characterization of T-DNA and CRISPR mutants for *AtPFK4* and *AtPFK5*. Figure S3: Alignment of plant phosphofructokinase isoenzymes. Figure S4: Significance of the remaining conserved Cys for redox regulation. Figure S5: Representative pictures of five-week-old *pfk5-CR1*, *pfk5-CR2*, *pfk5-2* and wildtype Col-0 plants. Figure S6: Expression atlas of *AtPFK5* from the eFP browser. Figure S7: Sugar content in leaves of five-week-old *pfk5-CR1*, *pfk5-CR2*, *pfk5-2* and wildtype Col-0 plants. Table S1: Primer sequences (a) and protein IDs (b) used in this study.
