# Supplementary material for: The Phosphofructokinase Isoform AtPFK5 Is a Novel Target of Plastidic Thioredoxin-f-Dependent Redox Regulation
Source: Antioxidants (Basel). 2021 Mar 7;10(3):401. doi: 10.3390/antiox10030401 (PMC7998735; doi:10.3390/antiox10030401)
Supplement: Supplementary file 1 [file antioxidants-10-00401-s001.pdf]

The phosphofructokinase isoform AtPFK5 is a novel target of plastidic thioredoxin-f-dependent redox regulation

Natalia Hess, Simon Richter, Michael Liebthal, Karl-Josef Dietz, Angelika Mustroph

SUPPLEMENTARY DATA

Supplementary Figures

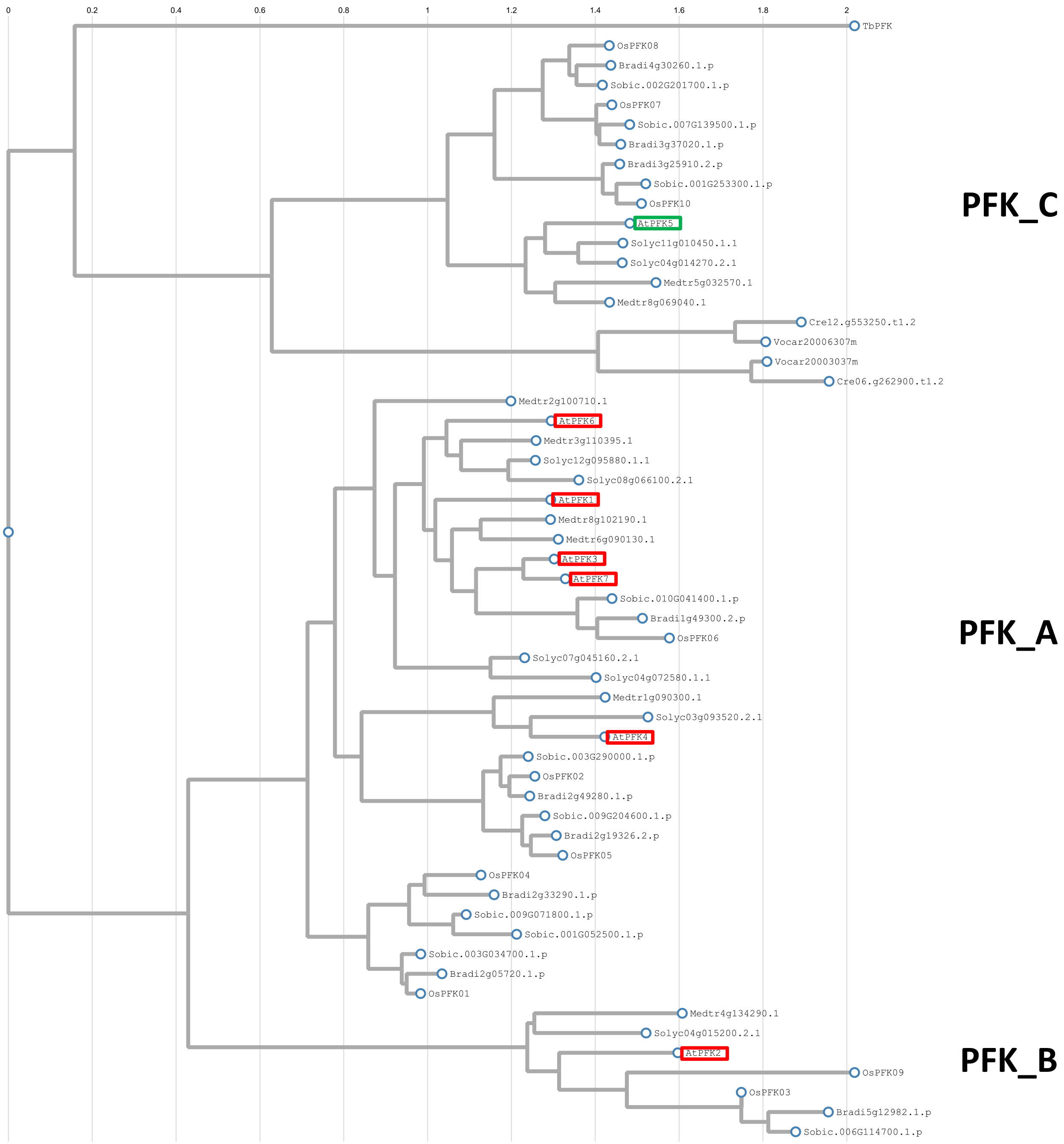

Supplementary Figure S1: Phylogeny of plant phosphofructokinase isoenzymes. PFK protein sequences were obtained from Phytozome and aligned using ClustalW using the platform https://www.genome.jp/tools-bin/clustalw#clustalw.aln. The phylogenetic tree was constructed with function "build" of ETE3 v3.1.1 (Huerta-Cepas et al., 2016) as implemented on the GenomeNet (https://www.genome.jp/tools/ete/). Species are abbreviated as follows: At, *Arabidopsis thaliana*; Bradi, *Brachypodium distachyon*; Cre, *Chlamydomonas reinhardtii*; Medtr, *Medicago truncatula*; Os, *Oryza sativa*; Sobic, *Sorghum bicolor*; Soly, *Solanum lycopersicum*; Tb, *Trypanosoma brucei*; Vocar, *Volvox carteri*. Protein IDs are found in Supplementary Table S1b. PFKs from Arabidopsis are highlighted in red, the target isoform of this study, AtPFK5, is highlighted in green.

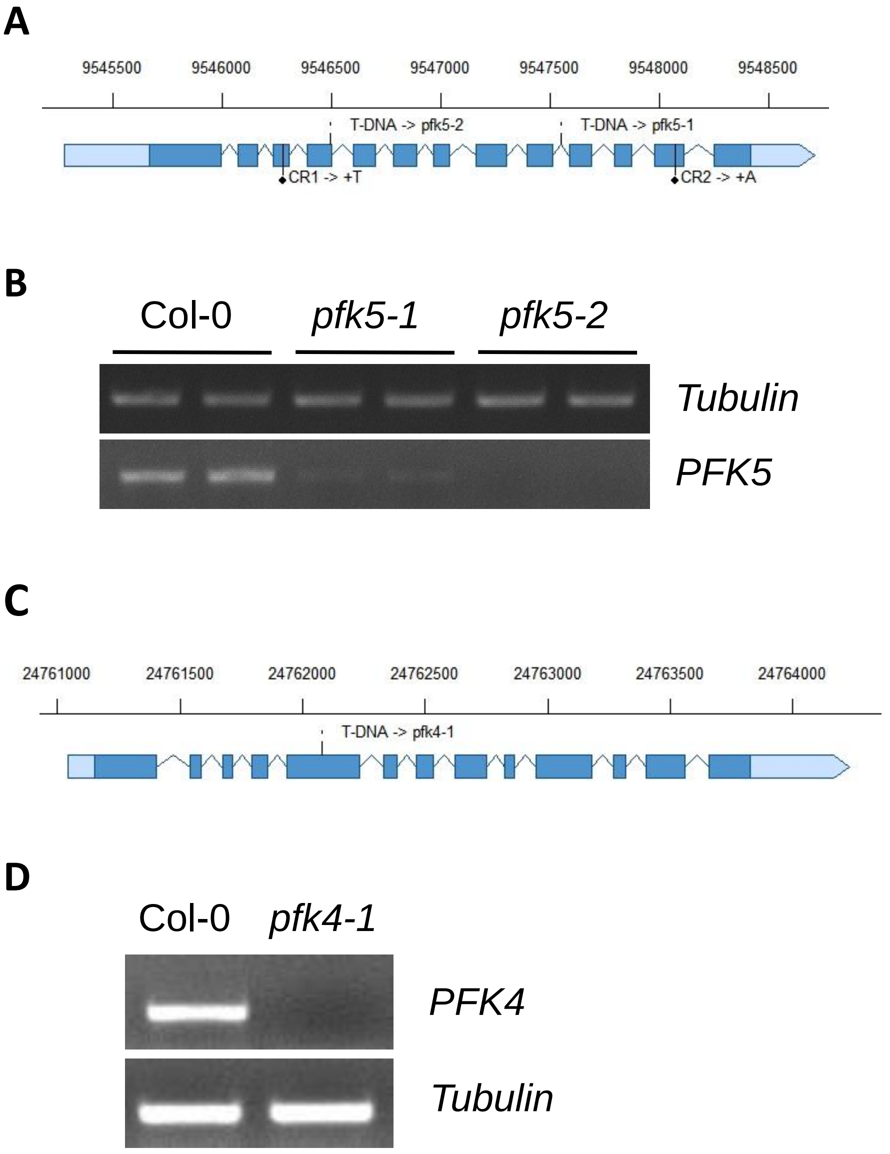

Supplementary Figure S2: Genotype characterization of T-DNA and CRISPR mutants for AtPFK4 and AtPFK5. **(A, C)** Gene models for *AtPFK5* and *AtPFK4*, including site of mutations. **(B, D)** Transcript levels of *AtPFK5* and *AtPFK4* in wildtype Col-0 and T-DNA insertion mutant seedlings. Tubulin was used as the reference.

Supplementary Figure S3 (continued over the next pages): Alignment of plant phosphofructokinase isoenzymes, which is the basis for the phylogenetic tree presented in Supplementary Fig. S1. Cys residues are in red font, and conserved motifs are marked with asterisks above the alignment.

```
Solyc04g014270.2.1   1  ---------------MESLSPAIGQKAIFPSTTGYT-----DHDLFLDSVKVRTERNFNRACRRLRIRVQ 50
Solyc11g010450.1.1   1  ---------------MESLSPAIGSNPIRPTTTISG-----ENELLISSVKSIKLPPVARRKMRMRVAAQ 50
AtPFK5               1  ---------------MDALSQAISSGISVPYKNNSSSLVPSHGLTSLILRKSRSPVNPSSRSRVSVRASE 55
Medtr5g032570.1      1  --------------------MNTHVITPNLNTTLLHHHHHHVHGLTNSSHRYSKTNHRLFHSFKVSNNVR 50
Medtr8g069040.1      1  ------------------------MASISHAITTTTNPYFNLPHQTQTPSSILTLSHSNSRRVFKNVGVF 46
OsPFK07              1  --------------------MAVSLKSSGSFCST-PPQWLHSTRDRILYGYSHSNAKECTCKKTKRPAPL 49
Bradi3g37020.1.p     1  --------------------MAVALKTIGSFCNT-QQQCLHSTRDRILYGYSHSNTKECKSKNAKRPAPL 49
Sobic.007G139500.1.p 1  --------------------MASALKTNGSFCSTQQQQFLQSTTDQFLHGSSHLNFKHCKSKKTIKPAPL 50
Sobic.002G201700.1.p 1  --------------------MTLSGMAVAFKASTSSTTQQHWP-SPTKDRCQYGFTQLSRQKYRKKFAAL 49
Bradi4g30260.1.p     1  -------------------MSSSPAMAIAFKASTSYFTAQQLCGHSTWDQCQNGFTHLNERKSRKGSAAL 51
OsPFK08              1  --------------------MTFSGMDIALKASTHSSTSQQHWLHSTRYRCQYGLGSTHLNGRKRSPMVL 50
OsPFK10              1  -----------------------MALKSPVDFAGSITSGQKDPCCFGVPGCN-PRCVRYNKKSRT-CRLV 45
Sobic.001G253300.1.p 1  ------------------------MALAPMDYAGSISSGQKYLGRFGVPTSSRLRWVGYDTKSRT-YQLV 45
Bradi3g25910.2.p     1  -----------------------MAIASPVDYAGPITSGQRRLSCFPVHSCNHLRCNAYEKKSRTSDQLV 47
Cre06.g262900.t1.2   1  ------------------------MRLQRHAAAGLGATKHRDTLTARLPCQSTRGRAALQVACVATPHSA 46
Vocar20003037m       1  ------------------------MRLQRLARTNASLGGQRRLCVGPGPCRSAPGRTRMQVSCVAETSET 46
Cre12.g553250.t1.2   1  ----------------------------------------------MLLQRHAPGFTKVPSRQCKPSVPI 24
Vocar20006307m       1  --------------------------------------------MRLQQRQVIGRSLPVCVPCVTRCRKL 26
Medtr6g090130.1      1  ---------------------------------------------------------------------- 1
Medtr8g102190.1      1  ---------------------------------------------------------------------- 1
AtPFK3               1  ---------------------------------------------------------------------- 1
AtPFK7               1  ---------------------------------------------------------------------- 1
Sobic.010G041400.1.p 1  ---------------MAPPPASPADSAPDPRPIASAPVPITVPSLRDHTHHHLVDRRDTPRVRAWDWEPE 55
Bradi1g49300.2.p     1  ----------------MASPSRTTDAATDPAPTPAPIAVPSLRARLPHAHHHLLDRRDTPRRPAWWSERA 54
OsPFK06              1  ---------------MASPPTASASASEAAESGRRSAPGPIDVPSPRDHLHHLLDRRDTPRVVHVEGTTM 55
AtPFK1               1  ---------------------------------------------------------------------- 1
Solyc08g066100.2.1   1  ---------------------------------------------------------------------- 1
Solyc12g095880.1.1   1  ---------------------------------------------------------------------- 1
Medtr3g110395.1      1  ---------------------------------------------------------------------- 1
AtPFK6               1  ---------------------------------------------------------------------- 1
Solyc04g072580.1.1   1  ---------------------------------------------------------------------- 1
Solyc07g045160.2.1   1  ---------------------------------------------------------------------- 1
OsPFK01              1  ---------------------------------------------MEAVGVAPAPAGVPEKKLLEVKES- 24
Sobic.003G034700.1.p 1  ---------------------------------------------MEAVGVAPAPVAAPEKKKLLDLKDP 25
Bradi2g05720.1.p     1  -----------------------------------MESVVVAPAAAAQGAVAVAEQQQQKKKLLDQKMEL 35
OsPFK04              1  ----------------------------------------MEAATVVAAPIPAADAAAKALEKKLLDLEL 30
Bradi2g33290.1.p     1  ----------------------------------------------MEAAQTVVLASVPASTAGVPQHKI 24
Sobic.001G052500.1.p 1  --------------------------------------------METATVVVAPAPVAITQPP-QQLMEL 25
Sobic.009G071800.1.p 1  --------------------------------------------METATVVVAPAPVSITQPPQHKLMEL 26
Medtr2g100710.1      1  ---------------------------------------------------------------------- 1
OsPFK02              1  -MASHIILPKEEE-----------AALGVAVEEDHDSPAAPGYQHQQGPPVAKALPFSATCVRISRDSYP 58
Bradi2g49280.1.p     1  -MASHIILPKEEEEE---------EGLGVAVEADHDSPEPP---RYQAEPVPKALPFSATCVRISRDSYP 57
Sobic.003G290000.1.p 1  MASSHIILPKEEEEEEEKEAAAVAEAGLGVGVEADHDSPAQR--PYQEQAPGKAALPFSATCVRISRDSYP 68
Sobic.009G204600.1.p 1  -MASHIILPPDDADENHNHQAEEEEREELGRDEDADDEEHG-HGHPPALPPKAALPFSATCVRISRDSYP 68
Bradi2g19326.2.p     1  -MASHIILPPDDEEQRQRDEEEDGGQ---------------------SASEHAPLPFSAMCVRISRDSYP 48
OsPFK05              1  MASSHIILPPDDDDEEQRRLEEEEDEDPWARSGPPP--------AAPEHAMKAALPFSATCVRISRDSYP 62
AtPFK4               1  ------------------------MEASISFLGSTKPNISLFNPSSNVLPRRDFPLPALKLKKVSVLPRI 46
Solyc03g093520.2.1   1  ---------------------MEFSLYNSSSLRFPQNDFLPLHNRSTLGSFHTFSIKKLASKSISTCRPL 49
Medtr1g090300.1      1  ---------------MDLSSSSSVYTSFSSLSKTPPSFSLSSSSSSSRQNSLQLLLLRSHLPQRALFLRN 55
Sobic.006G114700.1.p 1  -------------------------------------------------------------------MDP 3
Bradi5g12982.1.p     1  ---------------------------------------------------------------------- 1
OsPFK03              1  ---------------------------------------------------------------------- 1
AtPFK2               1  ---------------------------------------------------------------------- 1
Medtr4g134290.1      1  ---------------------------------------------------------------------- 1
Solyc04g015200.2.1   1  ---------------------------------------------------------------------- 1
OsPFK09              1  ---------------------------------------------------------------------- 1
TbPFK                1  ---------------------------------------------------------------------- 1
Clustal Consensus    1                                                                         1
```

```
Solyc04g014270.2.1     51  PKNAAAGVGGIDFSDPDWKLKYSREFEARFNIPHMTDVFPDAVSYPSTFCLKMRTPITEKFAEGYPSDEK 120
Solyc11g010450.1.1     51  SSNFVVPNN-IDFNDPDWKVKYEREFEARFNIPHITDLFPDAVSYPSTFCLKMRTPVTEDFAQGYPSDEE 119
AtPFK5                 56  IQHSKTSASSIDLSDPDWKLKYEKDFEQRFSIPHITDVLPDAEAIRSTFCLKMRS-PTEDFVGGYPSDEE 124
Medtr5g032570.1        51  VFSELKSQNKIDYNDPDWKDKFKEDFEARFRLPHITDIFPDASSMRSTFCLKMRAPITRDFHGNYDSGEE 120
Medtr8g069040.1        47  AEHRNSSSTSIDFNDPDWKFKFQQDFESRFRLPHITDIFPDSPPIPSTFCLRMRTPIGKDIPGHYTLDEE 116
OsPFK07                50  CVKATSTKVELDFNDPSWKQKFQEDWDKRFNLPRITDIYDLK-PRPTTFSLKKNRSPAGDENG--TPMDK 116
Bradi3g37020.1.p       50  CVKATSTKVELDFSDPSWKQKFQEDWDERFNLPRITDIYDDLKPRPTTFSLKKKRTPRSDEDS--TPMDM 117
Sobic.007G139500.1.p   51  CVRATSSKVELDFHDPSWKQKFQEDWERRFNLPSITDIYDLK-PRPTTFSLKKNRTLTGDEN-----VDM 114
Sobic.002G201700.1.p   50  HVTATSGKLDLDFTDPSWNQKYQEDWNRRFSLPHITDIYDLE-PRRTTFSLKKNRIPLGDGDG--SSADM 116
Bradi4g30260.1.p       52  HVRAISRNLDLDFSDPSWKQKYQEDWDMRFSLPHITDIFDLK-PRLTSFSLKKNRTPLGDNDG--SSPDM 118
OsPFK08                51  SVRAVSGKSDLDFSDPSWKEKYQEDWNRRFSLPHITDIYDLK-PRLTTFSLKKNRTDGGS-----LSADK 114
OsPFK10                46  TRAISVDRPQLDFSNSDWKKQFQEDFDRRFSLPHLKDVIDVE-PRPTTFSLKS-RTPLENVNG--SMQGS 111
Sobic.001G253300.1.p   46  ARAISVDQPQLDFSNPDWKKQFQEDFNKRFSLPHLRDVIDVE-PRPTTFSLKS-RTPLENVNG--TMQES 111
Bradi3g25910.2.p       48  IRAISVDRPQLDFSIPDWKNLFQEDFDRRFSLPHLTDVIHLE-PRPTTFSLKS-RTPLENGSG--PFDES 113
Cre06.g262900.t1.2     47  DSQKGAPKATATPSGQYVSSPYGAGRVLTPSPPGSIDDDDVLELKNLRNYLVPRDSPFIVDNN------Q 110
Vocar20003037m         47  AKQGPSPPPTLS-SGAYVTNSFGAGRVSRDS----LDEEDVLELKNLRNYLVPRDSPFIVDNN------H 105
Cre12.g553250.t1.2     25  ARQPRSSVCARATPGADLTTNYVIEPV-------SFGEDAVLECPEMRSKLVVRPSPFVTHNN------F 81
Vocar20006307m         27  ITVPRARPTPAATSGADLTTSYIIEPV-------SFGEDAVLECPDMRSKLQPRPSPFVVHNN------F 83
Medtr6g090130.1        1   ---------------MGLTEPKVVTGPAGYVLEDVPHLSDYIPDLP------TYPNPLQDNPA---YSVV 46
Medtr8g102190.1        1   ------------MGSSPNSKPKIVRGSAGYILEDVPHLTDYIPELP------TYSNPLQDNPA---YSVV 49
AtPFK3                 1   ------------MSTVESSKPKIINGSCGYVLEDVPHLSDYLPGLP------TYPNPLQDNPA---YSVV 49
AtPFK7                 1   ------------MSSPRSNKPKIVNGPGGYILQDVPHLIDYLPDLP------TYPNPLQDNPA---YSVV 49
Sobic.010G041400.1.p   56  RNRRG-------GAMDGAAAAKLVSGEAGYVLEDVPHVSDYLPDLP------TYPNPLQDNPA---YSVV 109
Bradi1g49300.2.p       55  TREAM-------DGTAAKPETKLVAGEAGYVLEDVPHLSDYLPDLP------TYSNPLQDNPA---YSVV 108
OsPFK06                56  QRQRGEAAGDAGAAAAAKPEVKLVTGDGGYVLEDVPHVCDYLPDLP------TYSNPLQDNPA---YSVC 116
AtPFK1                 1   -----------MSSSVPNSDRKIVTGPAGYILEDVPHFSDDFPDHP------TYPNPLQDNAA---YSVV 50
Solyc08g066100.2.1     1   ------------MGTESKYQMKVVNGDFGYVLEDVPHLTDYIPNIP------IFDNPLRSNPA---YSVV 49
Solyc12g095880.1.1     1   ------------MGTESNYQMKVVKGDHGYVLEDVPHLTDYIPDLP------THDNPLRSNPA---YSVV 49
Medtr3g110395.1        1   -----------MASSESNSQMKVVHGDAGYILEDVPHLSDYVPNLP------TYPNPLRSNPA---YSVV 50
AtPFK6                 1   -----------MASNGVDEQIKLVEGPAGYVLEDVPHLSDYILDLP------TYPNPLQSNAA---YSVV 50
Solyc04g072580.1.1     1   ------MGSLVDSFALHNLNKKIVMGDNGYVFEDVPHLSDYIPSLP------TYPNPLKKNPS---YSVV 55
Solyc07g045160.2.1     1   ---------MGEVTNSQQQKKKIVIGDDGYVLEDVPHLSDYIPNLP------TYTNPLQHNPS---YSVV 52
OsPFK01                24  -RKAAPAAPSTSMAAKWAMKKKLVGGDAGYVLEDVPHLTDYLPELP------TYPNPLQDNPA---YSVV 84
Sobic.003G034700.1.p   26  FPAAANGSAKASPAGKWAMKKKLVGGDAGYVLEDVPHLTDYMPHLP------TYPNPLQDNPA---YSVV 86
Bradi2g05720.1.p       36  PLVAALKAPAPASPCKWAMKKKLVGGDAGYVLEDVPHLTDYMPELP------TYPNPLQDNPA---YSVV 96
OsPFK04                31  PPFPAPAKKAAAKVVAAAPKKKLAGGAGGYVLEDVPHLTDYLPNLP------SFPNPLQNHPA---YSVV 91
Bradi2g33290.1.p       25  LDLKVMSADPAVQAPARKTKKKLSAGGGGYVLEDVPHLKDYLPHLP------SYPNPLQNHPA---YSVV 85
Sobic.001G052500.1.p   26  KFSFAPVAKSSPATKAEPDKKKLTGGCGGYVLEDVPHLTDYLPELK------SYPNPLQDHPA---YSVV 86
Sobic.009G071800.1.p   27  KPAFAAVAKPSPARKAKPAKKKLAGGCGGYVLEDVPHLTDYLPELK------SYPNPLQDHPA---YSVV 87
Medtr2g100710.1        1   ------------MVDSSTSHVEANTHVSDFLLEDVPHLSDYIPDLP------TYPDPLQDNPS---YSVV 49
OsPFK02                59  NLRALRNASAMSLPDDDAAYAKLEEGDYGYLLDDVPHFTDYLSDLP------TFPNPLQDHPA---YSTV 119
Bradi2g49280.1.p       58  NLRALRNASAVSLRDDDADFVKLEEGDYGYVLDDVQHLTDYLPELP------TFPNPLQDHPA---YSTV 118
Sobic.003G290000.1.p   69  NLRALRNASTMALHDDDAAFVKIEEGDFGYVLDDVPHLTDYLPDLP------TFPNPLQDHPA---YSTV 129
Sobic.009G204600.1.p   69  NLRALRNASSVSLAD--AAYVKISEGDYGYVLDDVPHFTDYLPDIP------TYPNPLQDHPA---YSTV 127
Bradi2g19326.2.p       49  NLRALRNASAVSLAD--AAYAKISEGDFGYVLDDVPHLADYLPDIP------TYPNPLQDHPA---YSTV 107
OsPFK05                63  NLRALRNASSVSLAD--AAYVKISEGDFGYVLDDVPHLVDHLPDAP------TYPNPLQDHPA---YSTV 121
AtPFK4                 47  LHQKRLIRAQCSDGFKPE-------EDDGFVLEDVPHLTKFLPDLP------SYPNPLKESQA---YAIV 100
Solyc03g093520.2.1     50  IWRRLPLVRRNAQTATESNASGDKHGEDDFIVEDVPHLTHFLPDLP------SYTNPLKKSQA---YAIV 110
Medtr1g090300.1        56  PILPPPFSRQNRLPLAPVRVSNQSENDDGFLLEDVPHLTNFLPNLP------SYPNPLQTSQA---YSIV 116
Sobic.006G114700.1.p   4   TAARSSGGTPAAAENDATAPTNTTVTLPPLTLRDVPRLPSALASAASPSPTPAVQNPISRHPY---FHPP 70
Bradi5g12982.1.p       1   ----MDPTTPSSGDHTASTPTNTTVTLPPLTLRDVPRLPAADP----------IPNPISSRPC---FHPP 53
OsPFK03                1   ---------------------------------------------------------------------- 1
AtPFK2                 1   --------------MAAETSIRKLPSLSGLRHR---------------------RNPLEDNPY---FHPS 32
Medtr4g134290.1        1   -----MDGSSSSSSSSSLELEDVTNLFEPIKLKDLPHLNHYISN------LKTFPNPLDQNPY---FHTP 56
Solyc04g015200.2.1     1   -----------------MSNSSPSISISNVQLQHISHLTNYNS------QIKTHTNPLAVNPF---FRTF 44
OsPFK09                1   ------------------MDDDGETPMPSLELHKLPTLAGAAIP-----------NPIARHPL---YHPS 38
TbPFK                  1   -----------MAVESRSRVTSKLVKAHRAMLNSVTQEDLKVDRLPG----ADYPNPSKKYSS------- 48
Clustal Consensus      1                                                                          1
```

```
                                                                          ******
Solyc04g014270.2.1    121 WHGYINNNDRVLLKVINYSSPTSAGA------------------ECIDSNCSWVEQWVHRAGPREKIFFK 172
Solyc11g010450.1.1    120 WHGYINNNDRVLLKTINYSSPTSAGA------------------ECIDPDCTWVEQWVHRAGPREKIYFK 171
AtPFK5                125 WHGYINNNDRVLLKVISYSSPTSAGA------------------ECLDHDCSWVEQWIHRAGPREKIYFR 176
Medtr5g032570.1       121 WNGYISDNDRVLLKTIYHSSPTSAGA------------------QCIDPGCNWVEQWVHRAGPRKNIYFK 172
Medtr8g069040.1       117 WNGYINNNDRVLLKTINYSSPKSAGA------------------ECIDPDCTWVEQWVHRAGPREKIYYK 168
OsPFK07               117 WNGYVNSDDRALLKVIKYSSPNSAGA------------------ECIDPDCSWVEQWVHRAGPRKEIYYE 168
Bradi3g37020.1.p      118 WNGYVNNDDRALLKVIKYSSPTSAGA------------------ECIDPDCSWVEQWVHRAGPRKEIYYE 169
Sobic.007G139500.1.p  115 WNGYVNNDDRALLKVIKYSSPTSAGA------------------ECIDPDCSWVEQWVHRAGPRKEIYYE 166
Sobic.002G201700.1.p  117 WNGYVNKSDRALLKVIKYASPTSAGA------------------LCIDPDCSWVEHWVHRAGPRKEIYYE 168
Bradi4g30260.1.p      119 WNGYVNKDDRALLKVIKYASPTSAGS------------------ECIDPDCSWVEHWVHRAGPRKEIYYE 170
OsPFK08               115 WNGYVNKDDRALLKVIKYASPTSAGA------------------ECVDPDCSWVEHWIHRAGPRKEIYYE 166
OsPFK10               112 WNGYVNDDDRALLKVIKFASPTSAGA------------------DCIDPDCSWVEQWVHRAGPRKQIYFE 163
Sobic.001G253300.1.p  112 WNGYVNDDDRALLKVIKFASPTSAGA------------------DCIDPDCSWVEQWVHRAGPRKQIYFE 163
Bradi3g25910.2.p      114 WNGYVNDDDRALLKVIKFASPTSAGA------------------DCIDPDCSWVEQWVHRAGPRKQIYFE 165
Cre06.g262900.t1.2    111 GGGFVGDRDRIRLHTVEFESTESAGS----------FCADGVLTNGDEDSCILLPEWAIRCGPRKTIYFD 170
Vocar20003037m        106 GGGFVGDRDRIRLHTVEFESAESAGS----------FCADGVLTHGDEDSCILLPEWAIRCGPRKTIYFD 165
Cre12.g553250.t1.2    82  GGGFVSDQDRVALNSMRFASPDSAGASRSNFFPHGGKGGINVLEASMDQLNMTLPPWAIRAGARREIYFD 151
Vocar20006307m        84  GGGFVSDNDRVALNSMRFASAESAGASRTSGEGQ-AKSSVNVLEASMDQLNMTLPPWAIRAGARKEIFFD 152
Medtr6g090130.1       47  KQYFVHVDDTVPQKVVVHKDSP-------------------------------RGVHFRRAGPRQKVYFE 85
Medtr8g102190.1       50  KQYFVHIDDSVPQKIIANKDSP-------------------------------RGIHFRRAGPRQKVYFK 88
AtPFK3                50  KQYFVDADDSVPQKIVVHKDGP-------------------------------RGIHFRRAGPRQKVYFE 88
AtPFK7                50  KQYFVHADDSVPEKVVVHKDGP-------------------------------RGVHFRRAGPRQKVYFE 88
Sobic.010G041400.1.p  110 KQYFVNPDDTVCQKIVVHKDGP-------------------------------RGNHFRRAGPRQRVYFE 148
Bradi1g49300.2.p      109 KQYFVNPDDTVCQKIVVHKGGA-------------------------------RGNHFRRAGPRQRVYFE 147
OsPFK06               117 RQYFVNPDDTVCQKAIVHKDGP-------------------------------RGNHFRRAGPRQRVFFE 155
AtPFK1                51  KQYFVDEDDTVPQKIVVHPDSP-------------------------------RGTHFRRAGPRQRVYFE 89
Solyc08g066100.2.1    50  KQYFVDMDDTVPQKIVVHKDSQ-------------------------------RGVHFRRAGPRQKVYFN 88
Solyc12g095880.1.1    50  KQYFVDMDDTVPQKVVVHKDSP-------------------------------RGVHFRRAGPRQKVYFN 88
Medtr3g110395.1       51  KQYFVHMDDTVPQKVVVHKDGP-------------------------------RGVHFRRAGPRQKVYFR 89
AtPFK6                51  RQYFVDEDDTVQEKIVVHKDSP-------------------------------RGTHFRRAGPRQKVYFK 89
Solyc04g072580.1.1    56  NQYFVDEDDTVAQNIVVRKNSP-------------------------------RGIHFRRAGPSQDVYFK 94
Solyc07g045160.2.1    53  KQYFVNEDDTVAEKVVVHKNSP-------------------------------RGIHFRRAGPCQKVYFE 91
OsPFK01               85  KQYFVNTDDTVTQKIVVHKTSA-------------------------------RGTHFRRAGPRQRVYFQ 123
Sobic.003G034700.1.p  87  KQYFVNPDDTVTQKIVVHKTSA-------------------------------RGTHFRRAGPRQRVYFQ 125
Bradi2g05720.1.p      97  KQYFVNTDDTVTQKIVVHKTSA-------------------------------RGTHFRRAGPRQRVYFQ 135
OsPFK04               92  KQYFVNADDTVAKKIVVHKGSA-------------------------------RGTHFRRAGPRQRVFFQ 130
Bradi2g33290.1.p      86  KQYFVNPGDTVSKKIVVHESSA-------------------------------RGTHFRRAGPRQRVYFQ 124
Sobic.001G052500.1.p  87  KQYFVKPDETVAKKVVVHKSSA-------------------------------RGTHFRRAGPRRRVYFQ 125
Sobic.009G071800.1.p  88  KQYFVNPDDTVAKKIVVHKSSA-------------------------------RGTHFRRAGPRQRVYFQ 126
Medtr2g100710.1       50  KQYYVHPDDTVAQQIVVHKNSP-------------------------------RGTLFRRAGPAQKVYFD 88
OsPFK02               120 KQYFVNADDTVPEKVVVQKDSP-------------------------------RGVHFRRAGPRQRVYFE 158
Bradi2g49280.1.p      119 KQYFVNADDTVPEKVVVQKNSP-------------------------------RGVHFRRAGPRQRVYFE 157
Sobic.003G290000.1.p  130 KQYFVNADDTVPEKVVVQKNSP-------------------------------RGVHFRRAGPRQRVYFE 168
Sobic.009G204600.1.p  128 KQYFVNEDDTVPQKVVVQKNSR-------------------------------RGVHFRRAGPRQRIYFG 166
Bradi2g19326.2.p      108 KQYFVNEDDTVPQKVVVQKNSR-------------------------------RGVHFRRAGPRQRVYFE 146
OsPFK05               122 KQYFVNEDDTVPQKVVVQKNSR-------------------------------RGVHFRRAGPRQKVYFE 160
AtPFK4                101 KRTFVSSEDVVAQNIVVQKGSK-------------------------------RGVHFRRAGPRERVYFR 139
Solyc03g093520.2.1    111 KKTFVSPEDMVAKEIIVQKDSP-------------------------------RGIHFRRAGPREKVFFT 149
Medtr1g090300.1       117 KNTFVSPEDVVAQTIVVQKNSP-------------------------------RGVHFRRAGPREKVYFK 155
Sobic.006G114700.1.p  71  ATFYISPGDVTLRHAFFDLA----------------------------SAAPSPLVAYRRAGPRGEIAVD 112
Bradi5g12982.1.p      54  PTFYISPGDVSLRHAFFDLA----------------------------SGTPDPLVAYRRAGPRGSLAVD 95
OsPFK03               1   ---------------------------------------------------------------------- 1
AtPFK2                33  NGFYITPSDVILAQVAYDHS----------------------------AHSQS-RVAYHRAGPRREIMYE 73
Medtr4g134290.1       56  --------QLQHFYITNNNL----------------------------VFSHSTRFAYRTAGPRNNVFFN 90
Solyc04g015200.2.1    45  PGFFLTSSDIVLRHNVLDLSGT--------------------------FPSPNPPLAYHRAGPRNQILFD 88
OsPFK09               39  PSFFISPTDVVLRDILFDASPASAAG----------------------ERRRRHVAAYHRAGPRREVAFD 86
TbPFK                 49  RTEFRDKTDYIMYNPRPRDEPSSENP------------------------VSVSPLLCELAAARSRIHFN 94
Clustal Consensus     1                                                                          1
```

```
                                        *****
Solyc04g014270.2.1     173 PEEVKAAIVTCGGLCPGLNDVIRQIVITLEIYGVKK--IVGIPFGYRGFSEK-GLAEMPLSRKVVQNVHL 239
Solyc11g010450.1.1     172 PEEVKAAIITCGGLCPGLNDVIRQIVITLEIYGVKQ--IVGIPFGYRGFSNN-DLAEMPLSRKVVQNVHL 238
AtPFK5                 177 PEEVKAAIITCGGLCPGLNDVIRHIVITLEIYGVKN--IVGIPFGYRGFSDK-DLTEMPLSRKVVQNIHL 243
Medtr5g032570.1        173 PEEVKAAIVTCGGLCPGLNDVIRQIVITLEIYGVTE--IVGIPFGYRGFSDK-ELMEIPLSRKVVQNIHL 239
Medtr8g069040.1        169 PEDVKAAIVTCGGLCPGLNDVIRQIVITLEIYGVKK--IVGIPFGYRGFSDK-ELTEVPLSRKVVQNIHL 235
OsPFK07                169 PEEVKAAIVTCGGLCPGLNDVIRQIVFTLETYGVKN--IVGIPFGYRGFFEK-GLKEMPLSRHLVENINL 235
Bradi3g37020.1.p       170 PEEVKAAIVTCGGLCPGLNDVIRQVVFTLETYGVKN--IVGIPFGYRGFFEK-GLKEMPLSRDLVENINL 236
Sobic.007G139500.1.p   167 PAEVKAAIVTCGGLCPGLNDVIRQIVFTLETYGVKN--IVGIPFGYRGFFEK-GLKEMPLSRGLVENINL 233
Sobic.002G201700.1.p   169 PEEVKAAIVTCGGLCPGLNDVIRQIVFTLEIYGVKN--IVGIPFGYRGFFEK-GLKEMSLSRDVVENINL 235
Bradi4g30260.1.p       171 PEEVKAAIVTCGGLCPGLNDVIRQIVFTLEIYGVKN--IVGIQFGYRGFFEK-GLKEMPLSRDVVENINL 237
OsPFK08                167 PAEVKAAIVTCGGLCPGLNDVIRQIVFTLEIYGVKN--IVGIQFGYRGFFEK-GLKEMPLSRKVVENINL 233
OsPFK10                164 PQYVKAGIVTCGGLCPGLNDVIRQIVLTLEKYGVKN--IVGIQHGFRGFFED-HLAEVPLNRQVVQNINL 230
Sobic.001G253300.1.p   164 PQYVKAGIVSCGGLCPGLNDVIRQIVLTLEKYGVKN--IVGIQHGFRGFFED-HLSEVPLSRHVVQNINL 230
Bradi3g25910.2.p       166 PQSVKAGIVTCGGLCPGLNDVIRQIVLTLEKYGVKN--IVGIQHGFRGFFED-HLSEVPLNRHVVQNINL 232
Cre06.g262900.t1.2     171 PQQVSAAVVTCGGLCPGLNDVVQNIVYTLTDYGVPEDNILGIRYGLRGFYER-DAKPITLTRKYVDGIHL 239
Vocar20003037m         166 PKQVSAAVVTCGGLCPGLNDVVQNIVYTLTDYGVPEDNILGIRYGLRGFYER-DAKPITLTRKYVDGIHL 234
Cre12.g553250.t1.2     152 PSQTTAAIVTCGGLCPGLNDVVQGLVNKLTDYGVPEGKILGIKYGFRGFYDP-QVKPIVLSKRVVDGIQL 220
Vocar20006307m         153 PQQVTAAIVTCGGLCPGLNDVVQGLVNKLTDYGVPEGKILGIKYGFRGFYDP-SVKPVVLTKRVVDGIQL 221
Medtr6g090130.1        86  SDEIQAAIVTCGGLCPGLNTVIRELVCSLHHMYGVT-RVLGIEGGYRGFYA---RNTITLTPSSVNNIHK 151
Medtr8g102190.1        89  SDEVQAAIVTCGGLCPGLNTVIRELVCGLYHMYGVK-KVLGISGGYKGFYA---HNTISLTPKTVNDIHK 154
AtPFK3                 89  SDEVHACIVTCGGLCPGLNTVIREIVSSLSYMYGVK-RILGIDGGYRGFYA---KNTVSLDSKVVNDIHK 154
AtPFK7                 89  SDEVHACIVTCGGLCPGLNTVIREVVSSLSYMYGVK-RILGIDGGYRGFYA---KNTIPLNSKVVNDIHK 154
Sobic.010G041400.1.p   149 PDEVHACIVTCGGLCPGLNTVIREIVCGLSDMYGVT-KILGIQGGYRGFYA---RNTITLTPKSVNDIHK 214
Bradi1g49300.2.p       148 SDEVHACIVTCGGLCPGLNTVIREIVCGLSDMYGVT-KILGIQGGYRGFYA---CNTINLTPKSVNDIHK 213
OsPFK06                156 SDEVHACIVTCGGLCPGLNTVIREIVCGLYDMYGVS-RVLGIQGGYRGFYA---CNTIDLSPKSVNDIHK 221
AtPFK1                 90  SDDVLACIVTCGGLCPGLNTVIREIVCGLSYMYGVK-RILGIDGGYRGFYA---RNTIHLDLKTVNDIHR 155
Solyc08g066100.2.1     89  SDDVRACIVTCGGLCPGLNTVIREIVHSLDYMYGVD-KVLGIEGGYRGFYA---KNTINLTPKLVNDIHK 154
Solyc12g095880.1.1     89  SDDVRACIVTCGGLCPGLNTVIREIVHSLDYMYGVS-KVFGIDGGYRGFYS---KNIINLTPKTVNDIHK 154
Medtr3g110395.1        90  SDDVIACIVTCGGLCPGLNTVIREIVCGLSYMYGVD-KVLGIDGGYRGFYS---KNTITLTPKVVNDIHK 155
AtPFK6                 90  PSDVRACIVTCGGLCPGLNTVIREIVCGLHFMYGVT-EVIGVDCGFRGFYS---KNTVALTPKTVSDIHK 155
Solyc04g072580.1.1     95  ADDVNACIVTCGGLCPGLNTVIREIVCGLYSMYGVA-RVMGIDGGYRGFYS---KNTIPLTPNVVNDIHK 160
Solyc07g045160.2.1     92  PDDVYACIVTCGGLCPGLNTVIREIVCGLHYMYGVS-RVMGIDGGYRGFYS---KNTIPLTPKVVNNIHK 157
OsPFK01                124 SDEVNAAIVTCGGLCPGLNTVIRELVCGLYDMYGVT-SVVGIEGGYKGFYS---RNTVALTPKSVNDIHK 189
Sobic.003G034700.1.p   126 SDEVNAAIVTCGGLCPGLNTVIRELVCGLYDMYGVT-SVVGIEGGYKGFYS---KNTVPLTPKSVNDIHK 191
Bradi2g05720.1.p       136 SDEVNAAIVTCGGLCPGLNTVIRELVCGLCDMYGVT-SVVGIEGGYKGFYS---RNTVPLTPKSVNDIHK 201
OsPFK04                131 PDEVSAAIVTCGGLCPGLNTVIRELVCGLHDMYGVT-SVVGIEGGYRGFYA---RNTVELTPRSVNGIHK 196
Bradi2g33290.1.p       125 PGEVVAAIVTCGGLCPGLNTVIRELVCGLHDMYGVG-SVVGIEGGYKGFYA---RNTVELTPRSVNGIHK 190
Sobic.001G052500.1.p   126 SDEVRAAIVTCGGICPGLNTVIRELVCGLHDMYGVT-SIHGIEGGYKGFYA---RNTVELTPRSVNGIHK 191
Sobic.009G071800.1.p   127 PDEVTAAIVTCGGLCPGLNTVIRELVCGLHDMYGVS-SIYGIEGGYKGFYA---RNTVELTPRSVNDIHK 192
Medtr2g100710.1        89  SEEVYACVVTCGGLCPGLNTVIRELVCGLYHMYGVQ-KVLGIGGGYRGFYS---RNTIPLTPKIVNDIHK 154
OsPFK02                159 SEDVKACIVTCGGLCPGLNTVIRELVCGLSHMYNVN-DIFGIQNGYKGFYS---SNYLPMTPKSVNDIHK 224
Bradi2g49280.1.p       158 PEDVKACIVTCGGLCPGLNTVIRELVCGLSHMYNVN-DVFGIQNGYKGFYS---SNYLPMTPKSVNDIHK 223
Sobic.003G290000.1.p   169 SEEVKACIVTCGGLCPGLNTVIRELVCGLSHMYNVS-NVFGIQNGYKGFYS---SNYLPMTPKSVNDIHK 234
Sobic.009G204600.1.p   167 TDEVKACIVTCGGLCPGLNTVIRELVCGLSHMYNVS-SIFGIQNGYKGFYS---SNYLPLTPKSVNDIHK 232
Bradi2g19326.2.p       147 SDEVKACIVTCGGLCPGLNTVIRELVCGLSHMYNVN-KIYGIQNGYKGFYS---SNYLAVTPKTVNDIHK 212
OsPFK05                161 SDEVKACIVTCGGLCPGLNTVIRELVCGLAHMYNVS-KIYGIQNGYKGFYS---SNYLTLTPKSVDDIHK 226
AtPFK4                 140 SDEVKACIVTCGGLCPGINTVIREIVCGLNNMYGVN-NILGIQGGYRGFYS---KNTMNLTPKVVNDIHK 205
Solyc03g093520.2.1     150 PEEVRACIVTCGGLCPGINTVIREIVCGLKNMYGVD-DVLGIQGGYSGFYS---KNTLNLTTKVVNDIHK 215
Medtr1g090300.1        156 AEEVRACIVTCGGLCPGINTVIREIVFGLKNMYGVE-DILGIEEGYKGFYS---KNTMRLTPKVVSDIHK 221
Sobic.006G114700.1.p   113 PAAARAALVTCGGLCPGLNTVLRELVVGLHELYGVR-HVFGVAAGYRGFYGT-DEDHVRLDPAAVDDWHK 180
Bradi5g12982.1.p       96  PARARAALVTCGGLCPGLNTVLRELVVGLQELYGVR-DVFGVAAGYRGFYGA-DVDHVRLDPAAVDDWHK 163
OsPFK03                1   ------------------------------------------------------------------- 1
AtPFK2                 74  PSAVKAAIVTCGGLCPGMNTVIRELVVGLWELYGVR-EIYGIPAGYRGFY---SMKAVKLDPKAVHDWHK 139
Medtr4g134290.1        91  PSDVRAAIVTCGGLCPGLNTVIRELVVGLWDLYGVR-QIFGITAGYRGFYSS-STQPVQLNPNIVHHWHT 158
Solyc04g015200.2.1     89  PVTVRAAIVTCGGLCPGMNTVIRELVVGLWELYGVR-EIFGIKAGYRGFYS---YDPVRLNPKMVDDWHK 154
OsPFK09                87  PATVRAAIFTCGGLCPGTNTVVRELVVGLSELYGVRGGVFGVRNGYRGFYS---DEVVPLDPAAVEHWHK 153
TbPFK                  95  PTETTIGIVTCGGICPGLNDVIRSITLTGINVYNVK-RVIGFRFGYWGLSKKGSQTAIELHRGRVTNIHH 163
Clustal Consensus      1                                                                       1
```

```
Solyc04g014270.2.1     240 SGGSLLGVSRGGPKVSDIVDSIQERGINMLFVLGGNGTHAGANAIHDECRKRR-IKAAVVGVPKTIDNDI 308
Solyc11g010450.1.1     239 SGGSLLGVSRGGPKVSDIVDSIQERGINMLFVIGGNGTHAGANAIHDECRRRR-MQVAVVGVPKTIDNDI 307
AtPFK5                 244 SGGSLLGVSRGGPSVSEIVDSMEERGINMLFVLGGNGTHAGANAIHNECRKRK-IKVAVVGVPKTIDNDI 312
Medtr5g032570.1        240 SGGSLLGVSRGGPGVSEIVDSLEERGINMLFVLGGNGTHAGANAIHDECCKRR-IKVSVIGVPKTIDNDI 308
Medtr8g069040.1        236 SGGSLLGVSRGGPGVSDIVDSLEDRGINMLFVLGGNGTHAGANAIHNECCKRR-LKVSVIGVPKTIDNDI 304
OsPFK07                236 AGGSFLGVSRGGAKTSEIVDSIQARRIDMLFVLGGNGTHAGANAIHEECRKRK-LKVSVVAVPKTIDNDI 304
Bradi3g37020.1.p       237 AGGSFLGVSRGGAKTSEIVDSIQARRIDMLFVLGGNGTHAGANAIHDECRKRK-LKVSVVAVPKTIDNDI 305
Sobic.007G139500.1.p   234 NGGSFLGVSRGGAKTSEIVDSIQARRIDMLFVLGGNGTHAGANAIHEECRKRK-LKVSVVAVPKTIDNDI 302
Sobic.002G201700.1.p   236 SGGSFLGVSRGGAKTSEIVDSIQARRIDMLFVIGGNGSHAGANAIHEECRKRK-LKVSVVAVPKTIDNDI 304
Bradi4g30260.1.p       238 SGGSFLGVSRGGAKTSEIVDSIQARRIDMLFVIGGNGSHAGAKAIHEECRKRK-LKVSVVAVPKTIDNDI 306
OsPFK08                234 SGGSFLGVSRGGAKTSEIVDSIQARRIDMLFVIGGNGSHAGANAIHEECRKRK-LKVSVVAVPKTIDNDI 302
OsPFK10                231 AGGSFLGVSRGGANISDIVDSIQARRLDMLFVLGGNGTHAGANLIHEECRKRK-LKVSIVGVPKTIDNDI 299
Sobic.001G253300.1.p   231 AGGSFLGVSRGGANISDIVDSIQARRLDMLFVLGGNGTHAGANAIHDECRKRK-LQVSIVCVPKTIDNDI 299
Bradi3g25910.2.p       233 SGGSFLGVSRGGASVSDIVDSIQARRLDMLFVLGGNGTHAGANAIHEECRKRK-LKVSIVGVPKTIDNDI 301
Cre06.g262900.t1.2     240 KGGTMLGTSRGGANVKEIVRRIDLWGLNMVFVVGGNGGNAAANAISEECEAQG-VCCSVVGVPKSIDNDI 308
Vocar20003037m         235 KGGTMLGTSRGGANVKEIVRRIDLWGLNMVFVVGGNGGNAAANAISDECEAQG-VVCTVVGVPKSIDNDI 303
Cre12.g553250.t1.2     221 QGGTILGTSRGGANIREIVKRIDMWGIDMLFVVGGNGGNAGANAINAMCRQHD-VPCSVVGVPKSIDNDI 289
Vocar20006307m         222 QGGTILGTSRGGANIREIVKRIDMWGIDMLFVVGGNGGNAGANAINAMCRQHD-VPCTVVGVPKSIDNDI 290
Medtr6g090130.1        152 RGGTILGTSRGGHDTNKIVDSIQDRGINQVYIIGGDGTQRGASVIFEEVRRRG-LKCAVVGIPKTIDNDI 220
Medtr8g102190.1        155 RGGTILGSSRGGHDTTKIVDSIQDRGINQVYIIGGDGTQRGAYRIFEEIRNRG-LKVAVVGIPKTIDNDI 223
AtPFK3                 155 RGGTILGTSRGGHDTTKIVDSIQDRGINQVYIIGGDGTQRGASVIFEEIRRRG-LKVAVIGIPKTIDNDI 223
AtPFK7                 155 RGGTIIGTSRGGHDTNKIVDSIQDRGINQVYIIGGDGTQRGASVIFEEIRRRR-LKVAVVGIPKTIDNDI 223
Sobic.010G041400.1.p   215 RGGTILGSSRGGHDTVKIVDSIQDRGINQVYVIGGDGTQRGAGVIFEEVRRRG-LKVSVAGIPKTIDNDI 283
Bradi1g49300.2.p       214 RGGTILGSSRGGHDTMKIVDSIQDRVINQVYVIGGDGSQRGARVIFEEVRRRG-LKVAVAGIPKTIDNDI 282
OsPFK06                222 RGGTVLGTSRGGHDTMKIVDSIQDRGINQVYVIGGDGTQRGAGVIFEEIRRRG-LKVAVAGIPKTIDNDI 290
AtPFK1                 156 SGGTILGTSRGGHNTTKIVDSIQDRGINQVYIIGGDGSQKGAAAIFEEIRKRK-LKVAVAGIPKTIDNDI 224
Solyc08g066100.2.1     155 RGGTILGTSRGGHDTKKIVDSIQDHGINQVYIIGGDGTLKGAAIIYEEIRRRG-LKVAVAGIPKTIDNDI 223
Solyc12g095880.1.1     155 RGGTILGSSRGGHDTTKIVDSIQDRGINQVYIIGGDGTQKGAAVIYEEIRRRD-LKVIVAGIPKTIDNDI 223
Medtr3g110395.1        156 RGGTILGTSRGGHDTGKIVDSIQDRGINQVYIIGGDGTQRGAAVIYEEVRRRG-LKVAIAGIPKTIDNDI 224
AtPFK6                 156 RGGTILGTSRGGHDTSKIVDNIQDREINQVYIIGGDGTQKGANAIYKEIRRRG-LKVAVAGIPKTIDNDI 224
Solyc04g072580.1.1     161 RGGTILGSSRGGHVTKKIVDSIQDRGINQVYIIGGDGTLKGASLIFEEITRRG-LNVVVVGIPKTIDNDI 229
Solyc07g045160.2.1     158 RGGTILGTSRGGHVTKKIVDSIQDRGINQVYIIGGDGTQRGAAVIFEEIRRRG-LNVAVAGIPKTIDNDI 226
OsPFK01                190 RGGTVLGTSRGGHDTGKIVDSIKDRGINQVYIIGGDGTQKGASVIYEEVRRRG-LKCSVVGVPKTIDNDI 258
Sobic.003G034700.1.p   192 RGGTVLGTSRGGHDTAKIVDCLQDRGINQVYIIGGDGTQKGASVIYEEVRRRG-LKCSVVGIPKTIDNDI 260
Bradi2g05720.1.p       202 RGGTILGTSRGGHDTAKIVDSLQDRGINQVYIIGGDGTQQGASVIYEEVRRRG-LKCSVVGVPKTIDNDI 270
OsPFK04                197 RGGTVLGTSRGGQDTGKIVDSIQDRGINQVYIIGGDGTQKGAATIHAEVQRRG-LKCAVVGVPKTIDNDI 265
Bradi2g33290.1.p       191 RGGTVLRASRGGQDTAKVVDSIQDRGINQVYIIGGDGTQKGAAQVHEEVQRRG-LKCAVVGVPKTIDNDI 259
Sobic.001G052500.1.p   192 RGGTLLGSSRGGQDTAKIVDSIQDRGFNQVYIVGGDGSQKGAASIHEEVQRRG-LKCAVVGVPKTIDNDI 260
Sobic.009G071800.1.p   193 RGGTVLGTSRGGQDTAKIVDSIQDRGVNQVYIIGGDGTQKGAASIHEEVQRRG-LKCAVVGVPKTIDNDI 261
Medtr2g100710.1        155 RGGTVLGTSYGGHDTSKIVDSIQDRGINQVYILGGFGTQNEAAMIFEEIRRRD-LKVAVVGIPKTIDNDI 223
OsPFK02                225 RGGTVLGTSRGGHDTKKIVDNIQDRGINQVYIIGGDGTQKGAYEIYKEIRRRG-LKVAVAGVPKTIDNDI 293
Bradi2g49280.1.p       224 RGGTVLGTSRGGHDTRKIVDNIQDRGINQVYIIGGDGTQKGAYEIFKEIRRRG-LKVAVAGIPKTIDNDI 292
Sobic.003G290000.1.p   235 RGGTVLGTSRGGHDTKKIVDNIQDRGINQVYIIGGDGTQKGAYEIYKEIRRRG-LHVAVAGIPKTIDNDI 303
Sobic.009G204600.1.p   233 RGGTVLGTSRGGHDTKKIVDNIQDRGINQVYIIGGDGTQKGAYEIFKEIRKRG-LKVSVAGVPKTIDNDI 301
Bradi2g19326.2.p       213 RGGTVLGTSRGGHDTKKIVDNIQDRGINQVYIIGGDGTQKGAYEIYKEIRKRG-LKVAVAGVPKTIDNDI 281
OsPFK05                227 RGGTVLGTSRGGHDTKKIVDNIQDRGINQVYIIGGDGTQKGAYEIFKEIRKRG-LKVSVAGIPKTIDNDI 295
AtPFK4                 206 RGGTFLQTSRGGHDTAKIVDNIQDRGINQVYIIGGGGTQKGAEKIYEEVERRG-LQVAVSGIPKTIDNDI 274
Solyc03g093520.2.1     216 RGGTFLQTSRGGHDTKKIVDNIQDRGINQVYIIGGDGTQKGAAAIYKEVEKRG-IKVAVAGIPKTIDNDI 284
Medtr1g090300.1        222 LGGTFLRTSRGGHDTHKIVDNIEDRGINQVYIIGGDGTQKGAARIYEEVEKRR-LQVAVAGIPKTIDNDI 290
Sobic.006G114700.1.p   181 KGGTVLKTTRGGFDLGKIVDGIVARGYTQIYAIGGDGTMRGAVAIFQEFKRRG-LNISITGIPKTVDNDI 249
Bradi5g12982.1.p       164 KGGTALKTTRGGFDLDKIVDGIVARGYTQVYAIGGDGTMRGAVAIFEEFKRRG-LHISITGIPKTVDNDI 232
OsPFK03                1   --------------------------------------MRGAVAIFNEFKRRG-LNISITGIPKTVDNDI 31
AtPFK2                 140 KGGTVLATSRGGFHLQKIVDAIHLNGYNQVYIIGGDGTMRGAVEIFKEISLRK-LEVGITVIPKTVDNDV 208
Medtr4g134290.1        159 KGGTFLQTSRGGFDLCKIVDAIQNQAFNQVYIIGGDGTMRGAVKIFNEIKQRK-LNVAVVGIPKTVDNDV 227
Solyc04g015200.2.1     155 RGGTVLETSRGGFDLKKIVDAIQDHGFNQLYIIGGDGTMRGAVEIFEELKQRK-LYISVVGIPKTVDNDV 223
OsPFK09                154 AGGAALGTSRGGFDLARIVDAIERHGFNQVYAVGGDGTMRGAARIHREVRRRGRLAVAVAGIPKTVDNDV 223
TbPFK                  164 YGGTILGSSRGPQDPKEMVDTLERLGVNILFTVGGDGTQRGALVISQEAKRRG-VDISVFGVPKTIDNDL 232
Clustal Consensus      1                                           *  :      :  :   :  :**::***: 14
```

```
Solyc04g014270.2.1     309 MLMDKTFGFDTAVEEAQRAINSAYIEAHSAYRGIGIVKLMGRSSGFIAMQASLASGQIDICLIPEVPFNL 378
Solyc11g010450.1.1     308 MLMDKTFGFDTAVEEAQRAINSAYIEAHSAYHGIGIVKLMGRSSGFIAMHASLASGQIDICLIPEVPFNL 377
AtPFK5                 313 LHMDKTFGFDTAVEEAQRAINSAYIEAHSAYHGIGVVKLMGRNSGFIAMQASLASGQVDICLIPEVPFNL 382
Medtr5g032570.1        309 LLMDKTFGFDTAVEEAQRAIYSAYIEAHSAYHGIGIVKLMGRSSGFIAMQSSLASGQIDVCLIPEVPFDL 378
Medtr8g069040.1        305 LLMDKTFGFDTAVEEAQRAINSAYIEAHSAYHGIGVVKLMGRSSGFIAMQASLSSGQVDICLIPEVPFNL 374
OsPFK07                305 LLMDKTFGFDTAVEEAQRAINSAYIEARSAYHGIGLVKLMGRSSGFIAMHASLSSGQVDVCLIPEVPFTL 374
Bradi3g37020.1.p       306 PLMDKTFGFDTAVEEAQRAINSAYIEARSAYHGIGLVKLMGRSSGFIAMHASLSSGQVDICLIPEVPFTL 375
Sobic.007G139500.1.p   303 LLMDKTFGFDTAVEEAQRAINSAYIEARSAYHGIGLVKLMGRSSGFIAMHASLSSGQVDVCLIPEVPFTL 372
Sobic.002G201700.1.p   305 LFMDKTFGFDTAVEEAQRAINSAYIEARSAYHGIGLVKLMGRSSGFIAMHASLSSGQIDVCLIPEVSFTL 374
Bradi4g30260.1.p       307 LFMDKTFGFDTAVEEAQRAINSAYIEARSAYHGVGLVKLMGRSTGFIAMHASLSSGQIDVCLIPEISFTL 376
OsPFK08                303 LFMDKTFGFDTAVEEAQRAINSAYIEARSAYHGIGLVKLMGRSSGFIAMQASLSSGQIDVCLIPEVSFTL 372
OsPFK10                300 LLMDKTFGFDTAVEAAQRAINSAYIEAHSAFHGIGLVKLMGRSSGFITMHASLSSGQVDICLIPEVPFTL 369
Sobic.001G253300.1.p   300 LLMDKTFGFDTAVEAAQRAINSAYIEAHSAFHGIGLVKLMGRSSGFITMQASLSSGQVDICLIPEVPFTL 369
Bradi3g25910.2.p       302 LLMDKTFGFDTAVEAAQRAINSAYIEAHSAFHGIGLVKLMGRSSGFITMHASLSSGQVDICLIPEVPFTL 371
Cre06.g262900.t1.2     309 LIIDKCFGFETAVQEAQRALLAAKVEAGSARNGLGVVKLMGRQSGFIAMQAAMASGVADVCLIPEIPFRM 378
Vocar20003037m         304 LLIDKCFGFETAVEEAQRALLAAKVEAGSARNGLGLVKLMGRQSGFIAMQASMASGVADVCLIPEIPFRM 373
Cre12.g553250.t1.2     290 LLIDKCFGFDTAVEESQRALMAAKVEASSARKGIGLVKLMGRQSGFIAMQASMASGVVDACLIPEVNFKL 359
Vocar20006307m         291 LLIDKCFGFDTAVEESQRALLAAKVEASSARKGIGLVKLMGRQSGFIAMQASMASGVVDACLIPELNFKL 360
Medtr6g090130.1        221 PIIDKSFGFDTAVEEAQRAINAAHVEAESFENGIGVVKLMGRYSGFIAMYATLASRDVDCCLIPESPFYL 290
Medtr8g102190.1        224 PVIDKSFGFDTAVEEAQRAINAAHVEAESVENGIGVVKLMGRYSGFIAMYATLANRDVDCCLIPETPFFL 293
AtPFK3                 224 PVIDKSFGFDTAVEEAQRAINAAHVEAESIENGIGVVKLMGRYSGFIAMYATLASRDVDCCLIPESPFYL 293
AtPFK7                 224 PVIDKSFGFDTAVEEAQRAINAAHVEAESNENGIGFVKLMGRYSGYIAMYATLASRDVDCCLIPESPFYL 293
Sobic.010G041400.1.p   284 PVIDKSFGFDTAVEEAQRAINAAHVEAVSAENGIGLVKLMGRHSGFIAHYATLASRDVDCCLIPESPFYL 353
Bradi1g49300.2.p       283 PVIDKSFGFDSAVEEAQRAINAAHVEAESAENGIGLVKLMGRYSGFIAHYATLASRDVDCCLIPESPFYL 352
OsPFK06                291 PVIDRSFGFDTAVEEAQRAINAAHVEAGSAENGIGLVKLMGRHSGFIAHYATLASRDVDCCLIPESPFYL 360
AtPFK1                 225 PIIDRSFGFDTAVEEAQRAINAAHVEATSFENGIGLVKLMGRYSGFIAMHATLASRDVDCCLIPESPFFL 294
Solyc08g066100.2.1     224 PIIDKSFGFDTAVEEAQRAINAAHVEAQSAENGIGVVKLMGRYSGFIAMYATLASRDVDCCLIPESPFFL 293
Solyc12g095880.1.1     224 PVIDKSFGFDTAVEEAQRAINAAHVEAESAENGIGVVKLMGRYSGFIAMYATLASRDVDLCLIPESPFYL 293
Medtr3g110395.1        225 PVIDRSFGFDTAVEEAQRAINAAHVEAESVENGIGVVKLMGRYSGFIAMHATLASRDVDCCLIPESPFYL 294
AtPFK6                 225 PVIDKSFGFDTAVEEAQRAINAAHVEATSVENGIGIVKLMGRYSGFIAMYATLASRDVDCCLIPESPFYL 294
Solyc04g072580.1.1     230 PVIDKSFGFDSAVEEAQRAISAAHVEATSFENGIGVVKLMGRDSGFIAMYASLASRDVDCCLIPESCFYL 299
Solyc07g045160.2.1     227 PVIDKSFGFDSAVEEAQRAISAAHVEATSFENGIGLVKLMGRDSGFIAMYATLASRDVDCCLIPESPFYL 296
OsPFK01                259 AVIDKSFGFDTAVEEAQRAINAAHVEAESAENGIGVVKLMGRNSGFIAMYATLASRDVDCCLIPESPFYL 328
Sobic.003G034700.1.p   261 AVIDKSFGFDTAVEEAQRAINAAHVEAESAENGIGVVKLMGRNSGFIAMYATLASRDVDCCLIPESPFYL 330
Bradi2g05720.1.p       271 AVIDKSFGFDTAVEEAQRAINAAHVEAESAENGIGVVKLMGRNSGFIAMYATLASRDVDCCLIPESPFYL 340
OsPFK04                266 AVIDRSFGFDTAVEEAQRAINAAHVEAESAENGVGVVKLMGRNSGFIAMYATLASRDVDLCLIPESPFYL 335
Bradi2g33290.1.p       260 AVIDKSFGFDTAVEEAQRAIDAAHVEAESAENGIGVVKLMGRNSGFIAMHATLASRDVDCCLIPESPFYL 329
Sobic.001G052500.1.p   261 AVIDKSFGFDTAVEAAQRAIDAAHVEAECGENGIGVVKLMGRNSGFIAMHATLASRDVDCCLIPESPFYL 330
Sobic.009G071800.1.p   262 AVIDKSFGFDTAVEEAQRAINAAHVEAESAENGIGVVKLMGRNSGFIAMYATLASRDVDCCLIPESPFFL 331
Medtr2g100710.1        224 PVIDKSIGFDTAVEEAQRAINSAHVEAESAENGIGVVKLMGRCSGFIAMYATLASRDVDCCLIPESPFYL 293
OsPFK02                294 AVIDKSFGFDSAVEEAQRAIDAAHVEASSAENGIGLVKLMGRYSGFIAMYATLASRDVDCCLIPESPFYL 363
Bradi2g49280.1.p       293 AVIDKSFGFDTAVEEAQRAIDAAHVEASSAENGIGLVKLMGRYSGFIAMCATLASRDVDCCLIPESPFYL 362
Sobic.003G290000.1.p   304 AVIDKSFGFDTAVEEAQRAINAAHVEASSAENGIGLVKLMGRYSGFIAMYATLASRDVDCCLIPESPFYL 373
Sobic.009G204600.1.p   302 AIIDKSFGFDTAVEEAQRAIDSAHVEACSAENGIGLVKLMGRYSGFIAMYATLASRDVDCCLIPESPFYL 371
Bradi2g19326.2.p       282 AIIDKSFGFDSAVEEAQRAIDSAHVEACSAENGIGLVKLMGRYSGFIAMYATLASRDVDCCLIPESPFYM 351
OsPFK05                296 AIIDKSFGFDTAVEEAQRAIDSAHVEACSAENGIGLVKLMGRYSGFIAMYATLASRDVDCCLIPESPFYM 365
AtPFK4                 275 AVIDKSFGFDTAVEEAQRAINAAHVEVESVENGVGIVKLMGRYSGFIAMIATLANRDVDCCLIPESPFFL 344
Solyc03g093520.2.1     285 AVIDKSFGFDTAVEEAQRAINAAHVEAESVQNGVGIVKLMGRYSGFIAMLATLGNRDVDCCLIPESPFYL 354
Medtr1g090300.1        291 AVIDKSFGFDTAVEEAQRAINAAHVEVNSVENGIGIVKLMGRYSGFIAMNATLASRDVDCCLIPESPFYL 360
Sobic.006G114700.1.p   250 GIIDRSFGFQTAVEIAQQAIDAAHVEAVSAVNGVGLVKLMGRSTGHIALHATLSSRDVDCCLIPEVDFHL 319
Bradi5g12982.1.p       233 GIIDRSFGFQTAVEVAQQAINAAHVEAVSAVNGIGLVKLMGRSTGHIALYATLSSRDVDCCLIPEIDFHV 302
OsPFK03                32  GIIDRSFGFQTAVEIAQQAIDAAHVEAVSAVNGIGLVKLMGRSTGHIALHATLSSRDVDCCLIPEVDFYL 101
AtPFK2                 209 GIIDRSFGFQTAVEMAQEAISAAHVEAESAVNGIGLVKLMGRSTGHIALHATLSSRDVDCCLIPEMDFYL 278
Medtr4g134290.1        228 GIIDRSFGFQTAVEMAQQAISAAHVEAESAVNGIGLVKLMGRSTGHIVLHSTLSSRDVDCCLIPELDFYL 297
Solyc04g015200.2.1     224 GIIDRSFGFQTAVEMAQQAINAAHVEAESAVNGIGLVKLMGRSTGHIALHATLSSRDVDCCLIPEIDFYL 293
OsPFK09                224 GVVDRSFGFHTAVEAAQQAIAAGHVEAESAANGVGLVKLMGRSAGHIALHATLSSRDVDCCLIPEEDFYL 293
TbPFK                  233 SFSHRTFGFQTAVEKAVQAIRAAYAEAVSANYGVGVVKLMGRDSGFIAAQAAVASAQANICLVPENPISE 302
Clustal Consensus      14     .: :**.:**: : .*: :.  *. .   *:*.****** :*.*.  ::..   : **:**  :   49
```

```
Solyc04g014270.2.1      379 HGPHGVLRHLKYLLETKGSAVVCAAEGAG------QDFLEKTNAKDAS---------GNAVLGDIGVYLQ 433
Solyc11g010450.1.1      378 HGPHGVLQHLKYLLETKGSAVICVAEGAG------QDFLEKTNAKDAS---------GNAVLGDIGVHIQ 432
AtPFK5                  383 HGPNGVLKHLKYLIETKGSAVICVAEGAG------QNFLEKTNAKDAS---------GNAVLGDFGVYIQ 437
Medtr5g032570.1         379 HGPHGVLSHLKYLIESKGSAVVCVAEGAG------QNLLQKTNDTDPS---------GNAKLGDIGVYIQ 433
Medtr8g069040.1         375 HGPHGVLRHLQYLLEMKGSAVVCVAEGAG------QNLLQNTNAKDAS---------GNIVFGDIGVYIQ 429
OsPFK07                 375 DGEYGVLRHLEHLLKTKGFCVVCVAEAAGQLFYVHYRSLQKSGATDAS---------GNVILSDIGVHMQ 435
Bradi3g37020.1.p        376 DGEYGVLQHLEHLLENKGFCVVCVAEAAG------QELLQKSGATDAS---------GNVILSDIGVHMQ 430
Sobic.007G139500.1.p    373 DGEFGVLQHLEHLLKSKGFCVVCVAEAAG------QDLLQKSGATDAS---------GNVIFSDIGVHMQ 427
Sobic.002G201700.1.p    375 DGEHGVLRHLEHLLNTKGFCVVCVAEGAG------QDLLQKSNATDAS---------GNVILSDFGVHMQ 429
Bradi4g30260.1.p        377 DGERGVLAHLEHLLKTKGFCVVCVAEGAG------QDLLQKSNATDAS---------GNVILSDFGVHMQ 431
OsPFK08                 373 DGEHGVMRHLEHLLEKKGFCVVCVAEGAG------QDLLQKSNATDAS---------GNVILSDFGVHMQ 427
OsPFK10                 370 DGPNGVLQHLEHLIETKGFALICVAEGAG------QEHLQQSNATDAS---------GNMILGDIGVHLH 424
Sobic.001G253300.1.p    370 DGPNGVLRHLEHLIETKGFALVCVAEGAG------QEYFQKSNATDAS---------GNMVLSDIGVHLQ 424
Bradi3g25910.2.p        372 DGPNGVLRHLEHLIDTKGFALICIAEGAG------QEYLQKSNATDAS---------GNMVLSDIGAHLQ 426
Cre06.g262900.t1.2      379 DK---LCEHVESIFEKQGHCVVCVAEGAG----QDLLTAGGTGGTDAS---------GNPILADIGIFMR 432
Vocar20003037m          374 DK---LIAHIASVFEKQGHCVVCVAEGAG----QDLLLNG-AAGTDAS---------GNPILADIGIFLR 426
Cre12.g553250.t1.2      360 DGDNGLLKYLDGVIKAKGHAVVCVAEGAG----QDILEDGGQIGTDAS---------GNPILKDIGAFLK 416
Vocar20006307m          361 NGDQGLLRYLEGVIKNKGHCVVCVAEGAG----QDLLEDGGQLGTDAS---------GNPILKDIGAFLK 417
Medtr6g090130.1         291 EGPGGLYEYIDRRLKENGHMVIVIAEGAG------QELLTESLQSGKK----Q-DASGNKLLQDVGLWIS 349
Medtr8g102190.1         294 EGPGGLFEYTERRLKENGHMVIVIAEGAG------QELVTESIQSMHK----QQDASGNKLLQDVGLWIS 353
AtPFK3                  294 EGEGGLFEYIEKRLKESGHMVLVIAEGAG------QDLMSKSMESMTL----KDAS-GNKLLKDVGLWLS 352
AtPFK7                  294 EGEGGLFEFIERRLKDHGHMVIVLAEGAG------QDLMCKSMESTPM----DAS--GNKLLKDVGLWLS 351
Sobic.010G041400.1.p    354 EGEGGLFRYIEKRLKDNGHMVIVVAEGAG------QKLIAETMQSIGK----DAS--GNELLLDVGLWLS 411
Bradi1g49300.2.p        353 EGEGGLYRYIEKRLKDNGHMVIVVAEGAG------QKLFAETMKAMGQ----DAS--GNALLLDVGLWLS 410
OsPFK06                 361 EGEGGLFRYLEKRLKENGHMVIVVAEGAG------QKLINETKESMGK----DAS--GNSILLDVGLWLS 418
AtPFK1                  295 EGSGGLFEFIDKRLKESGHMVIVIAEGAG------QDLLSESMKESTT----LKDASGNKLLQDIGLWIS 354
Solyc08g066100.2.1      294 DGSGGLFEFVKKRLREEGHMVIVIAEGAG------QELLAAENSNAGS----QQDASGNKLLQDVGLWVS 353
Solyc12g095880.1.1      294 EGDGGLFEYIEKRLKENGHMVIVIAEGAG------QELLAEENAHAKN----EQDASGNKLLQDVGLWIS 353
Medtr3g110395.1         295 EGKGGLYEFITKRLKESGHMVIVIAEGAG------QDLLTESMQDMD-----QKDASGNKLLQDVGLWIS 353
AtPFK6                  295 EGKGGLYEFIAKRLRENGHMVIVIAEGAG------QDLVAES----IE----QQDASGNKLLKDVGLWMS 350
Solyc04g072580.1.1      300 EGRGGLFEYIEQRLKENGHMVIVVAEGVG------QDDE----------------------QVDVGLWIS 341
Solyc07g045160.2.1      297 EGSGGLFEYIEHRLKDNGHMVIVIAEGAG------QELVSESLKSTDP----SGNK----LLQDVGLWIS 352
OsPFK01                 329 EGKGGLLEFIEKRLKDNGHMVIVVAEGAG------QDLIAKSMNFVDT----QDAS-GNKLLLDVGLWLS 387
Sobic.003G034700.1.p    331 EGKGGLLEFIEKRLKDNGHMVIVVAEGAG------QDLIAKSMNFVDT----QDAS-GNKLLLDVGLWLS 389
Bradi2g05720.1.p        341 EGKGGLLEFIEKRLKDSGHMVIVVAEGAG------QDLIAQSMNFVDT----QDAS-GNKLLLDVGLWLS 399
OsPFK04                 336 EGKGGLLEFAEKRLRENGHMVIVVAEGAG------QDVIARSMRLADA----HDAS-GNKVLLDVGLWLC 394
Bradi2g33290.1.p        330 EGKDGLLEFCEKRLRENGHMVIVVAEGAG------QDLIAKGMHRADT----HDAS-GNRHLLDVGLWLS 388
Sobic.001G052500.1.p    331 EGKGGLLEFVEKRLRDNGHMVIVVAEGAG------QDLVAKGMPFANT----HDAS-GNKVLLDVGLWLS 389
Sobic.009G071800.1.p    332 DGKGGLLEFVEKRLRDNGHMVIVVAEGAG------QDLIAKSMNFADT----HDAS-GNKVLLDVGLWLS 390
Medtr2g100710.1         294 EGPGGLLEFIEKRLREQGHMVIVIAEGAG------QELIPRNDTSNKN----KPDASPDDLVQDVGLWLS 353
OsPFK02                 364 EGEGGLFEYIEKRLKENNHMVIVVAEGAG------QDLIAKSIAAADQ----IDAS-GNKLLLDVGLWLT 422
Bradi2g49280.1.p        363 EGEGGLFEYIERRLKENNHMVIVVAEGAG------QDLIAKSIPAADQ----LDAS-GNKLLLDVGLWLT 421
Sobic.003G290000.1.p    374 EGEGGLFEYIDRRLKENNHMVIVVAEGAG------QDLIAQSIPAADQ----QDAS-GNKLLLDVGLWLT 432
Sobic.009G204600.1.p    372 DGEGGLLQYVEKRLKENKHMVIVVAEGAG------QDLIAKSIATSDQ----QDAS-GNKLLLDIGLWLT 430
Bradi2g19326.2.p        352 DGEGGLLQYIEKRLKENKHMVIVVAEGAG------QDIIAKSIPSSDQ----QDAS-GNKLLLDIGLWLT 410
OsPFK05                 366 DGEGGLLQYVERRLKENKHMVIVVAEGAG------QDLIAKSLSTSEQ----QDAS-GNKLLLDIGLWLT 424
AtPFK4                  345 EGKGGLFEFIEERLKENRHMVIVIAEGAG------QDYVAQSMRASET----KDAS-GNRLLLDVGLWLT 403
Solyc03g093520.2.1      355 EGPGGLYEYVEQQLKENRHVLIVLAEGAG------QEYVAQSMQAFQE----KDAS-GNRLLLDVGLWLT 413
Medtr1g090300.1         361 EGRGGIFEFIEQRLKENGHLVIVVAEGAG------QECVASEVNTVDE----KDAS-GNKLLLDVGPWLS 419
Sobic.006G114700.1.p    320 EGKGGLFEFLYERIKKKGHAVIVVAEGAG------QELIPRTDDQKRE-----QDESGNIVFLDVGPWLK 378
Bradi5g12982.1.p        303 EGKGGLFEFLYERIKQKGHAVVVVAEGAG------QDLIPRTDDQKRE-----QDESGNNLFLDIGPWLK 361
OsPFK03                 102 EGKGGLFEFLYERIKQKGHAVVVVAEGAG------QELIPRTDDQKRE-----QDESGNIVFLDVGPWLK 160
AtPFK2                  279 EGKGGLFEFLEKRLKERGHAVLVVAEGAG------QEMIPRNESQKQE-----RDESGNAVFLDVGVWFK 337
Medtr4g134290.1         298 EGKGGLFEFLDQRLKENGHAVLVVAEGAG------QDIIPRTESHKEE-----RDESGNPVFLDVGVWLK 356
Solyc04g015200.2.1      294 EGKGGLFEFLEDRLKEMGHAVLVVAEGAG------QDIIPRTESQKTE-----KDESGNPVFLDVGGWLK 352
OsPFK09                 294 RGAGGLFDFLYRRIKDNGHAVVVVAEGAG------QRLIPRTTTTSASGACAGADESGNETFLDVGAWLK 357
TbPFK                   303 QE---VMSLLERRFCHSRSCVIIVAEGFG------QDWGRGSGGYDAS---------GNKKLIDIGVILT 354
Clustal Consensus       49       :       :      ::  **. *                                  *.*  .   58
```

```
Solyc04g014270.2.1    434 QEVKKYFKE---LGQTADVKYIDPTYMIRACRANASDGILCTVLGQNAVHGAFAGYSGITVGICNTHYVY 500
Solyc11g010450.1.1    433 QQIKKYFKE---IGVTTDVKYIDPTYMIRACRANASDGILCTVLGQNAVHGAFAGYSGITVGICNTHYVY 499
AtPFK5                438 QETKKYFKE---ISTPIDVKYIDPTYMIRAVRANASDGILCTVLGQNAVHGAFAGYSGITVGIINTHYAY 504
Medtr5g032570.1       434 QETKNYFKE---NDIHADVKYIDPTYMIRACRANASDGILCTVLGQNAVHGAFAGYSGITVGICNTHYAY 500
Medtr8g069040.1       430 QETKKYFKE---IGVHADVKYIDPTYMIRACRANASDGILCTVLGQNAVHGAFAGYSGISVGICNTHYAY 496
OsPFK07               436 QKIKMHFKD---IGVPADVKYIDPTYMVRACRANASDAILCTVLGQNAVHGAFAGFSGITSCICNTHYVY 502
Bradi3g37020.1.p      431 QKIKMHFKG---IGVHADVKYIDPTYMVRACRANASDAILCTVLGQNAVHAAFAGFSGITSCICNTHYVY 497
Sobic.007G139500.1.p  428 QKIKTHFKD---IGVPADVKYIDPTYMVRACRANASDAILCTVLGQNAVHGAFAGFSGITSCICNTHYVY 494
Sobic.002G201700.1.p  430 QKIKKHFKD---IGVPADLKYIDPTYMVRACRANASDAILCTVLGQNAVHGAFAGFSGITSGVCNTHYVY 496
Bradi4g30260.1.p      432 QKIKKHFKD---IGVLADIKYIDPTYMVRACRANASDAILCTVLGQNAVHGAFAGFSGITSGICNTHYAY 498
OsPFK08               428 QKIKSHFKD---IGVPADVKYIDPTYMVRACRANASDAILCTVLGQNAVHGAFAGFSGITSGICNTHYAF 494
OsPFK10               425 QKIKAHFKE---IGVHSDVKYIDPTYMVRAVRANASDAILCTVLGQNAVHGAFAGFSGITTGICNTHNVY 491
Sobic.001G253300.1.p  425 QKIKSHFKD---IGVHSDIKYIDPTYMLRAVRANASDAILCTVLGQNAVHGAFAGFSGITTGVCNTHNVY 491
Bradi3g25910.2.p      427 QKIKSHFNE---IGVHSDVKYIDPTYMLRAVRANASDAILCTVLGQNAVHGAFAGFSGITTGVCNTHNVY 493
Cre06.g262900.t1.2    433 NEFKKHFKG------EADIKYIDPSYMIRSVPTTSNDRIYCKVLGQGAVHGAFAGFTDVTVGLVNTHYVY 496
Vocar20003037m        427 NELKKHFKG------DADIKYIDPSYMIRSVPTTSNDRIYCKVLGQGAVHGAFAGYTDITVGLVNTHYVY 490
Cre12.g553250.t1.2    417 DKFKAYFK-------DADIKYIDPSYMIRSVSTTTNDRIYCKILAHNAVHAAFAGFTGITVGLVNTHYVY 479
Vocar20006307m        418 EKFKAYFK-------DADIKYIDPSYMIRSVSTTTNDRIYCKILAHNAVHAAFAGFTGITVGLVNTHYVY 480
Medtr6g090130.1       350 QSIKDHFAREK-TLPIT-LKYIDPTYMIRAVPSNASDNVYCTLLAQSAVHGAMAGYTGYTSGLVNGRQTY 417
Medtr8g102190.1       354 HKLKDYFAKQK-TMGIT-LKYIDPTYMIRAVPSNASDNVYCTLLAQSAVHGAMAGYTGFTSGLVHGRQAY 421
AtPFK3                353 QSIKDHFNQKK-MVMN--LKYIDPTYMIRAVPSNASDNVYCTLLAQSAVHGAMAGYTGYISGLVNGRQTY 419
AtPFK7                352 QSIKDHFKKNK-MVMN--LKYIDPTYMIRAVPSNASDNVYCTLLAQSAVHGAMAGYTGYTSGLVNGRQTY 418
Sobic.010G041400.1.p  412 QKINEYFKKNK-MTIN--LKYIDPTYMIRAIPSNASDNVYCTLLAHSVVHGAMAGYTGFTIGQVNGRHCY 478
Bradi1g49300.2.p      411 QKINEHFKKTK-TTIN--LKYIDPTYMIRAIPSNASDNVYCTLLAHSVVHGAMAGYTGFTVGQVNGRHCY 477
OsPFK06               419 QKIKEHFKKIK-TTIN--LKYIDPTYMIRAIPSNASDNVYCTLLAHSVVHGAMAGYTGFTVGQVNGRHCY 485
AtPFK1                355 QRIKDHFAK-K-MTLT--LKYIDPTYMIRAVPSNASDNVCCTLLAQSAVHGVMAGYNGFTVGLVNGRHTY 420
Solyc08g066100.2.1    354 HKIRDHFGKKL-KMPIT-LKYIDPTYMIRAVPSNASDNVYCTILAQSCVHGAMAGYTGFTSGIVNGRQTY 421
Solyc12g095880.1.1    354 QKIRDHFAKKT-KMPIT-LKYIDPTYMIRAVPSNASDNVYCTLLAQSCVHGAMAGYTGFTSGLVNGRQTY 421
Medtr3g110395.1       354 HKIKDHFAKEN-KMAIV-LKYIDPTYMIRAIPSNASDSVYCTLLAQSAVHGAMAGYTGFTAGLVNGRHTY 421
AtPFK6                351 LKIKEYFAKHN-VMDIT-LKYIDPTYMIRAIPANASDNVYSTLLAQSAVHGAMAGYTGFVSGLVNGRHTY 418
Solyc04g072580.1.1    342 TRIKEHFSKEK-KMMIN-LKYIDPTYMIRAIASNASDNMYCTHLAQSAVHGAMAGYTGFTVGPVNNTHAY 409
Solyc07g045160.2.1    353 ERIKEHFSKQK-KMLIN-LKYIDPTYMIRAIPSNASDNVYCTLLAQSAVHGAMAGYTGFTVGPVNNRNAY 420
OsPFK01               388 QKIKDHFKKKR-NFPIT-LKYIDPTYMIRAVRSNASDNVYCTLLAHSALHGAMAGYTGFTVAPVNGRHAY 455
Sobic.003G034700.1.p  390 QKIKDHFKKKP-SFPIT-LKYIDPTYMIRAVRSNASDNVYCTLLAHSALHGAMAGYTGFTVAPVNGRHAY 457
Bradi2g05720.1.p      400 QKIKDHFKKKT-NFPIT-LKYIDPTYMIRAVRSNASDNVYCTLLAHSALHGAMAGYTGFTVAPVNGRHAY 467
OsPFK04               395 AKIKDHFKKKA-NFPIT-LKYIDPTYMIRAVPSNASDNVYCSLLAHSAIHGAMAGYTGFTVAPVNGRHAY 462
Bradi2g33290.1.p      389 QKIKEHFKKKT-NLPIT-LKYIDPTYMIRAVPSNASDNVYCTLLAHSAVHGAMAGYTGFIVAPVNGRHAY 456
Sobic.001G052500.1.p  390 QQIKEHFKKKP-RFPIT-LKYIDPTYMIRAVPSNASDNVYCTLLAHSALHGAMAGYTGFSVGPVNGRHAY 457
Sobic.009G071800.1.p  391 HKIKEHFKKKP-SFPIT-LKYIDPTYMIRAVPSNASDNVYCTLLAHSALHGAMAGYTGFTVAPVNGRHAY 458
Medtr2g100710.1       354 QKIKDHFERSK-KIIN--LKYIDPTYMIRAIPSNASDNVFCTLLAQSAVHGAMAGYTGFTVGPVNGRNCY 420
OsPFK02               423 HKIKDYCKNKK-MEMT--IKYIDPTYMIRAIPSNASDNVYCTLLAHSAIHGAMAGYS-FTVGMVNGRHAY 488
Bradi2g49280.1.p      422 HKIKDYCKSKK-MEMT--IKYIDPTYMIRAIPSNASDNVYCTLLAHSAIHGAMAGYS-STVGMVHGRHAY 487
Sobic.003G290000.1.p  433 HKIKDYCKSKK-MEMT--IKYIDPTYMIRAIPSNASDNVYCTLLAHSAIHGAMAGYS-FTVGMVNGRHAY 498
Sobic.009G204600.1.p  431 HKIKDYFKSKK-MEMT--IKYIDPTYMIRAIPSNASDNVYCTLLAHSAIHGAMAGYS-FTVGMVNGRHTY 496
Bradi2g19326.2.p      411 HKIKDHFKSKK-VEMT--IKYIDPTYMIRAIPSNASDNVYCTLLAHSAIHGAMAGYS-FTVGMVNGRHTY 476
OsPFK05               425 HKIKDHFKSKK-MEMT--IKYIDPTYMIRAIPSNASDNVYCTLLAHSAIHGAMAGYS-FTVGNVNGRHAY 490
AtPFK4                404 QQIKDHFTNVR-KMMIN-MKYIDPTYMIRAIPSNASDNVYCTLLAQSAVHGAMAGYSGFTVGPVNSRHAY 471
Solyc03g093520.2.1    414 QQIKDHFTNER-KMTIN-MKYIDPTYMIRAIPSNASDNIYCTLLAQSAVHGAMAGFTGFTVGPVNSRHAY 481
Medtr1g090300.1       420 NKIKDHFTKLP-KMVVN-MKYIDPTYMIRAIPSNASDNIYCTLLAHSAVHGAMAGYSGFTVGPVNSKHAY 487
Sobic.006G114700.1.p  379 SELGRWWKREH-PDELFTVKYIDPTYMIRAVPANATDNLYCTLLAHSAIHGVMAGFTGFVPGPVNGTYSY 447
Bradi5g12982.1.p      362 SEVGRWWKREH-PSELFTLKYIDPTYMIRAVPANATDNLYCTLLAHSAIHGIMAGFTGFVPGPINGTYSY 430
OsPFK03               161 SELGKWWKREH-PSELFTVKYIDPTYMIRAVPANATDNLYCTLLAHSAIHGIMAGYTGFVPGPINGNYSY 229
AtPFK2                338 SVLKAWWEREH-PDELFTVKYIDPTYMIRAVPANATDNLYCTLLAHSAIHGVMAGYTGFVPGPINGNYAY 406
Medtr4g134290.1       357 SELNKWWDRDH-PGELFTVKYIDPTYMIRAVHANATDNLYCTLLAHSAIHGVMAGYTGFVAAPINGNYAY 425
Solyc04g015200.2.1    353 SELKNWWSRDH-PNELFTIKYIDPTYMIRAVPPNATDNSYCTLLAHSAIHGVMAGYTGFVCGPINGNYAY 421
OsPFK09               358 AEMRAWWEEEH-AGEVFTVKYIDPTYMIRAVPANAGDNLYCTLLAHAAIHGAMAGYTGFVSGTINGNYAY 426
TbPFK                 355 EKVKAFLKANKSRYPDSTVKYIDPSYMIRACPPSANDALFCATLATLAVHEAMAGATGCIIAMRHNNYIL 424
Clustal Consensus     58                    :*****:**:*:  ..: *   .  *.   :*  :** .       :      79
```

```
                                                         *
Solyc04g014270.2.1     501 FPIPEVISKPRVVDPNSRMWHRCLTSTGQPDFL------------------------------------- 533
Solyc11g010450.1.1     500 FPIPEVIAQPKVVDPNSRMWHRCLTSTGQPDFL------------------------------------- 532
AtPFK5                 505 LPITEVIAYPKSVDPNSRMWHRCLTSTGQPDFI------------------------------------- 537
Medtr5g032570.1        501 FPITEVISHPQLVDPNSRMWHRCLTSTGQPDFI------------------------------------- 533
Medtr8g069040.1        497 FPIPEVISHPRLVDPNSRMWHRCLTSTGQPDFI------------------------------------- 529
OsPFK07                503 LPITEVITVPKRVNPNSRMWHRCLTSTGQPDFH------------------------------------- 535
Bradi3g37020.1.p       498 LPITEVITSPKRVNPNSRMWHRCLTSTGQPDFH------------------------------------- 530
Sobic.007G139500.1.p   495 LPITEVITASKRVNPNSRMWHRCLTSTGQPDFH------------------------------------- 527
Sobic.002G201700.1.p   497 LPITEVITTPKHVNPNSRMWHRCLTSTGQPDFH------------------------------------- 529
Bradi4g30260.1.p       499 LPITEVITTPKHVNPNSRMWHRCLTSTGQPDFD------------------------------------- 531
OsPFK08                495 LPITEVITKPKRVNPNSRMWHRCLTSTGQPDFH------------------------------------- 527
OsPFK10                492 LPISEVIKSTRFVDPNSRMWHRCLTSTGQPDFH------------------------------------- 524
Sobic.001G253300.1.p   492 LPIPEVIKSTRFVDPNSRMWHRCLTSTGQPDFH------------------------------------- 524
Bradi3g25910.2.p       494 LPIPEVIKSTRFVDPNSRMWHRCLTSTGQPDFH------------------------------------- 526
Cre06.g262900.t1.2     497 LPIPTIIQAARKVNPKGRRWNRLITAIRQPDMA------------------------------------- 529
Vocar20003037m         491 LPIPMIIQAPRKVNPKGRRWNRLIAAIRQPDFS------------------------------------- 523
Cre12.g553250.t1.2     480 LPIPVIIQAPRKVDPRGKAWNRLRAAIGQPSFQ------------------------------------- 512
Vocar20006307m         481 LPIPVIIQAPRKVDPRGKAWNRLRAAIGQPSFT------------------------------------- 513
Medtr6g090130.1        418 IPFYRITERQNNVVITDRMWARLLSSTNQPSFMEVKVSDEDTKGENSLDELPDGHCSEDTSVDEITHNLG 487
Medtr8g102190.1        422 IPFNRINEGQNTVVITDRMWARLLSSTNQPSFLIAKTAVEEKKKEEEEEDSFNL---------------- 475
AtPFK3                 420 IPFYRITEKQNHVVITDRMWARLLSSTNQPSFLGPKDVFD-NKEKPMSALLDDGNCNGVVDVPPVTKEIT 488
AtPFK7                 419 IPFYRITETQNNVVITDRMWARLLSSTNQPSFLGPKDTSEEKKELPETPLLDDG----AVDIPPVTKEVT 484
Sobic.010G041400.1.p   479 IPFYRITEKQNRVSITDRMWARLLSSTNQPSFLCNKVVEEAKKEQERAAQLLDGSPSHRKG--------- 539
Bradi1g49300.2.p       478 IPFYRITEKQNKVSITDRMWARLLSSTNQPSFLCKQDVEDAKVEDERTAKLLDGSPSNPKVEDGLASSNS 547
OsPFK06                486 IPFYRITEKQNKVSITDRMWARLLSSTNQPSFLSKKDVEDAKMEEERASKFFDGPPPNPKVEDKVASNGK 555
AtPFK1                 421 IPFNRITEKQNKVVITDRMWARLLSSTNQPSFMKQADKIHSNQLVGEPGTMKW----------------- 473
Solyc08g066100.2.1     422 LPFNRITEKQNNVVITDRMWARLLASTNQPSFLSTNDIVQVHKRQHSQSQLLNGDENESEITGTYLKVLD 491
Solyc12g095880.1.1     422 IPFNRITEKQNMVVITDRMWARLLSSTNQPSFLRVKDIEEIKKEEQPQTQLLDGDNNVHENSGH------ 485
Medtr3g110395.1        422 IPFNRITEKMNNVVITDRMWARMLSSTNQPSFLKPKDVHQIMKAGQSPTQLLEGSNCNGGGEEAKKVEQI 491
AtPFK6                 419 IPFNRITERQNKVVITDRMWARMLSSTNQPSFMNPPKGTTEFTD-------------------------- 462
Solyc04g072580.1.1     410 IPFDRITEKQNKVDITDMMWAQLLSSTNQPSFLSTARSADNIEANKDEEPPIQLSDEETNQLSFLTTRVD 479
Solyc07g045160.2.1     421 IPFNRIIEKQNKVVITDRMWARLLSSTNQPSFLRTR--VDIIKANKDEEPPTQLSDDATTDDNLMEKQIL 488
OsPFK01                456 IPFYRITEKQNKVVITDRMWARVLCSTNQPCFLSHEDVEHLKHDDDE-HHLHNTQLLEGESSPVKDSSKC 524
Sobic.003G034700.1.p   458 IPFYRITEKQNKVVITDRMWARVLCSTNQPCFLSHEDVENMKHDDDE-HHLHNTQLLEGESSPVKDASKC 526
Bradi2g05720.1.p       468 IPFYRITEKQNKVVITDRMWARVLCSTNQPCFLSHEDVENMKHDDDEQHHLHNTQLLDG--SPAKCSPKC 535
OsPFK04                463 IPFYRITEKQNKVVITDRMWARVLCSTNQPCFLSTEDVEKAGQD----DEEPIVPLVEGENSLVKAPPLL 528
Bradi2g33290.1.p       457 IPFYRITEKQNKVVITDRMWARVLCSTNQPCFLSHEDVEKARQD----EEEPHVPLIEGENSLVRTSPMS 522
Sobic.001G052500.1.p   458 IPFYRITEKQNKVSITDRMWARVLCSTNQPCFLTEEDVEGAGLELEEEEPHMHMPLVEGGMQRLGQEQPV 527
Sobic.009G071800.1.p   459 IPFYRITEKQNKVVITDRMWARVLCSTNQPCFLTHEDVEGAAGLDEEEP---HMPLVEGEYALVRSPSMC 525
Medtr2g100710.1        421 IPFHLINEGQKRVVITDRMWARLLSSTHQPSFVNPQHITEEAKVE------------------------- 465
OsPFK02                489 IPFHRVTSTRNKVKITDRMWARLLSSTNQPSFLSQKDIDAAREADKLASKSPVPVNTKEHGENVKKPANG 558
Bradi2g49280.1.p       488 IPFHRVTSTRNKVKITDRMWARLLSSTNQPSFLTQKDIDEAREADRLANRPPVPAGTSELREHTEKHPAS 557
Sobic.003G290000.1.p   499 IPFHRVTSTRNKVRITDRMWARLLSSTNQPSFLSQKDIDAAREADKAANAKNQSAPALANGEK------- 561
Sobic.009G204600.1.p   497 IPFYRVTSTRNKVKVTDRMWARLLSSTNQPSFLSQKDIDEASEADRLANRPPLPTGASSRVARSFDQSSS 566
Bradi2g19326.2.p       477 IPFYRVTSTRNKVKITDRMWARLLSSTNQPSFLSQKDIDEAREADRLANKPPLPSGANQSVANAFGQSAS 546
OsPFK05                491 IPFYRVTSTRNKVKITDRMWARLLSSTNQPSFLSQKDIDEAKENDRTANKPPLPTGLSHHVANSFDQSAS 560
AtPFK4                 472 IPISQVTEVTNTVKLTDRMWARLLASTNQPSFLTGEGALQNVIDMETQEKIDNMKISSI----------- 530
Solyc03g093520.2.1     482 IPINRVTETTNTVNMTGRMWARLLASTNQPSFVN-HQTLRERVDKNTIDAINNMKINST----------- 539
Medtr1g090300.1        488 LPIACVTERPNTVQLTGRMWARLLESTNQPSFVS---SDQQRVEKEMVEDTNKMNITSI----------- 543
Sobic.006G114700.1.p   448 IPLEDVAVAKNPVDVNDHKWAWVRSVTNQPDFLKSQA--------------------------------- 484
Bradi5g12982.1.p       431 IPLEDIAVAKNPVDVNDHKWAWVRSVTDQPDFLKPQDY-------------------------------- 468
OsPFK03                230 IPLEDVAVAKNPVDVNDHKWAWVRSVTNQPDFMKPKY--------------------------------- 266
AtPFK2                 407 IPLEEVAQTKNQVNTRDHKWAWVRSVTNQPDFETNVKG-------------------------------- 444
Medtr4g134290.1        426 IPLEDVACAKNPVNTKDHNWSWVRSVTNQPDFLKS----------------------------------- 460
Solyc04g015200.2.1     422 IPVDEVAKAKNKVDTRDHKWSWVRSVSNQPDFIRS----------------------------------- 456
OsPFK09                427 IPMDEVAEAKNPVDTKDHKWAWVRSITNQPDFIRAGPTS------------------------------- 465
TbPFK                  425 VPIKVATSVRRVLDLRGQLWRQVREITVDLGSDVRLARKLEIRRELEAINRNRDRLHEELAKL------- 487
Clustal Consensus       79 .*.       . :   .  *        :                                          83
```

```
Solyc04g014270.2.1    533 ---------------------------- 533
Solyc11g010450.1.1    532 ---------------------------- 532
AtPFK5                537 ---------------------------- 537
Medtr5g032570.1       533 ---------------------------- 533
Medtr8g069040.1       529 ---------------------------- 529
OsPFK07               535 ---------------------------- 535
Bradi3g37020.1.p      530 ---------------------------- 530
Sobic.007G139500.1.p  527 ---------------------------- 527
Sobic.002G201700.1.p  529 ---------------------------- 529
Bradi4g30260.1.p      531 ---------------------------- 531
OsPFK08               527 ---------------------------- 527
OsPFK10               524 ---------------------------- 524
Sobic.001G253300.1.p  524 ---------------------------- 524
Bradi3g25910.2.p      526 ---------------------------- 526
Cre06.g262900.t1.2    529 ---------------------------- 529
Vocar20003037m        523 ---------------------------- 523
Cre12.g553250.t1.2    512 ---------------------------- 512
Vocar20006307m        513 ---------------------------- 513
Medtr6g090130.1       488 CLAPLAVPVLRTGGISGV---------- 505
Medtr8g102190.1       475 ---------------------------- 475
AtPFK3                489 K--------------------------- 489
AtPFK7                485 K--------------------------- 485
Sobic.010G041400.1.p  539 ---------------------------- 539
Bradi1g49300.2.p      548 NGVK------------------------ 551
OsPFK06               556 AVK------------------------- 558
AtPFK1                473 ---------------------------- 473
Solyc08g066100.2.1    492 LLPCLSCGQ------------------- 500
Solyc12g095880.1.1    485 ---------------------------- 485
Medtr3g110395.1       492 PTQLMEGDKSKDNQKSRNLADSDSCIKK 519
AtPFK6                462 ---------------------------- 462
Solyc04g072580.1.1    480 INEANKNKEPPTELSDGETN-------- 499
Solyc07g045160.2.1    489 TF-------------------------- 490
OsPFK01               525 NGTAAPV--------------------- 531
Sobic.003G034700.1.p  527 NGTV------------------------ 530
Bradi2g05720.1.p      536 NGFAAPL--------------------- 542
OsPFK04               529 ANAGDRAALCNGAA-------------- 542
Bradi2g33290.1.p      523 MCNGHGHF-------------------- 530
Sobic.001G052500.1.p  528 DVQRANGNGHL----------------- 538
Sobic.009G071800.1.p  526 NGNGHLCSGAA----------------- 536
Medtr2g100710.1       465 ---------------------------- 465
OsPFK02               559 EK-------------------------- 560
Bradi2g49280.1.p      558 ESANGEK--------------------- 564
Sobic.003G290000.1.p  561 ---------------------------- 561
Sobic.009G204600.1.p  567 CSSNGEI--------------------- 573
Bradi2g19326.2.p      547 SSSNGEI--------------------- 553
OsPFK05               561 SSSNSQI--------------------- 567
AtPFK4                530 ---------------------------- 530
Solyc03g093520.2.1    539 ---------------------------- 539
Medtr1g090300.1       543 ---------------------------- 543
Sobic.006G114700.1.p  484 ---------------------------- 484
Bradi5g12982.1.p      468 ---------------------------- 468
OsPFK03               266 ---------------------------- 266
AtPFK2                444 ---------------------------- 444
Medtr4g134290.1       460 ---------------------------- 460
Solyc04g015200.2.1    456 ---------------------------- 456
OsPFK09               465 ---------------------------- 465
TbPFK                 487 ---------------------------- 487
Clustal Consensus     83                                83
```

Supplementary Figure S4 (next page): Significance of the remaining conserved Cys for redox regulation. Cys to serine single and double mutations were introduced into *A. thaliana* AtPFK5 **(A)** and AtPFK3 **(B;C)**. The variants were transiently overexpressed in tobacco leaves. PFKs from the tobacco leaf samples were extracted with buffer containing 5 mM β-mercaptoethanol (β-ME). After initial activity determination, samples were oxidized with 2.5 mM sodium tetrathionate (TT) for 20 min, and afterwards re-reduced with 5 mM dithiotreitol (DTT). Upper case letters indicate significant differences for each isoform to buffer and GFP control, lower case letters indicate significant differences among treatments for each isoform at $p < 0.05$ (ANOVA, Post-hoc Tukey test, n=3-6). The results are means ± SD. **(D-F)** Details of the protein alignment of conserved Cys in PFKs from *A. thaliana* and *O. sativa*. The mutated Cys are marked with an arrow.

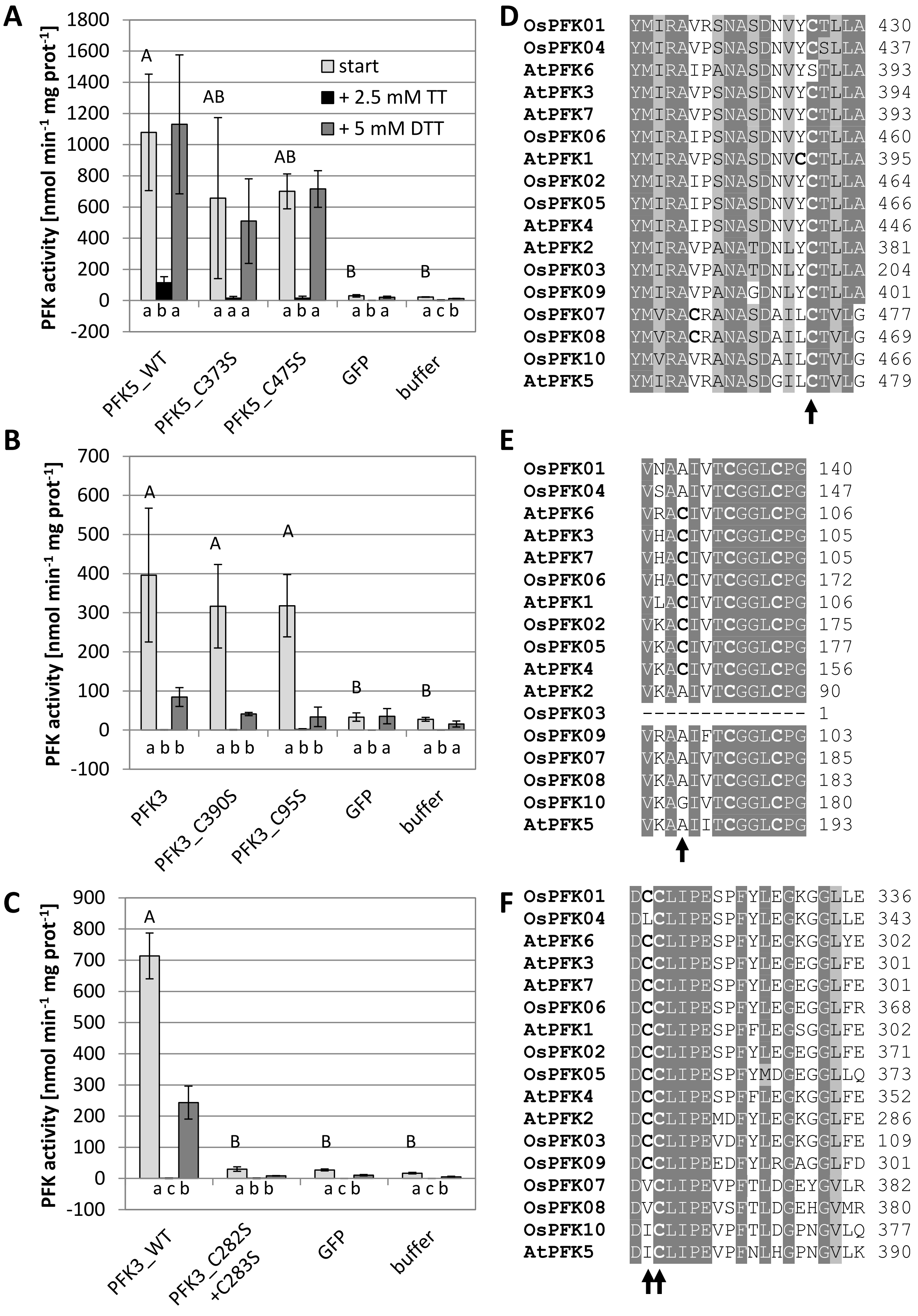
A
PFK activity [nmol min-1 mg prot-1]
start
+ 2.5 mM TT
+ 5 mM DTT
A
AB
AB
B
B
a b a
a a a
a b a
a b a
a c b
PFK5_WT
PFK5_C373S
PFK5_C475S
GFP
buffer
B
PFK activity [nmol min-1 mg prot-1]
A
A
A
B
B
a b b
a b b
a b b
a b a
a b a
PFK3
PFK3_C390S
PFK3_C95S
GFP
buffer
C
PFK activity [nmol min-1 mg prot-1]
A
B
B
B
a c b
a b b
a c b
a c b
PFK3_WT
PFK3_C282S +C283S
GFP
buffer
D
OsPFK01 YMIRAVRSNASDNVYCTLLA 430
OsPFK04 YMIRAVPSNASDNVYCSLLA 437
AtPFK6 YMIRAIPANASDNVYSTLLA 393
AtPFK3 YMIRAVPSNASDNVYCTLLA 394
AtPFK7 YMIRAVPSNASDNVYCTLLA 393
OsPFK06 YMIRAIPSNASDNVYCTLLA 460
AtPFK1 YMIRAVPSNASDNVCCTLLA 395
OsPFK02 YMIRAIPSNASDNVYCTLLA 464
OsPFK05 YMIRAIPSNASDNVYCTLLA 466
AtPFK4 YMIRAIPSNASDNVYCTLLA 446
AtPFK2 YMIRAVPANATDNLYCTLLA 381
OsPFK03 YMIRAVPANATDNLYCTLLA 204
OsPFK09 YMIRAVPANAGDNLYCTLLA 401
OsPFK07 YMVRACRANASDAILCTVLG 477
OsPFK08 YMVRACRANASDAILCTVLG 469
OsPFK10 YMVRAVRANASDAILCTVLG 466
AtPFK5 YMIRAVRANASDGILCTVLG 479
E
OsPFK01 VNAAIVTCGGLCPG 140
OsPFK04 VSAAIVTCGGLCPG 147
AtPFK6 VRACIVTCGGLCPG 106
AtPFK3 VHACIVTCGGLCPG 105
AtPFK7 VHACIVTCGGLCPG 105
OsPFK06 VHACIVTCGGLCPG 172
AtPFK1 VLACIVTCGGLCPG 106
OsPFK02 VKACIVTCGGLCPG 175
OsPFK05 VKACIVTCGGLCPG 177
AtPFK4 VKACIVTCGGLCPG 156
AtPFK2 VKAAIVTCGGLCPG 90
OsPFK03 -------------- 1
OsPFK09 VRAAIFTCGGLCPG 103
OsPFK07 VKAAIVTCGGLCPG 185
OsPFK08 VKAAIVTCGGLCPG 183
OsPFK10 VKAGIVTCGGLCPG 180
AtPFK5 VKAAIITCGGLCPG 193
F
OsPFK01 DCCLIPESPFYLEGKGGLLE 336
OsPFK04 DLCLIPESPFYLEGKGGLLE 343
AtPFK6 DCCLIPESPFYLEGKGGLYE 302
AtPFK3 DCCLIPESPFYLEGEGGLFE 301
AtPFK7 DCCLIPESPFYLEGEGGLFE 301
OsPFK06 DCCLIPESPFYLEGEGGLFR 368
AtPFK1 DCCLIPESPFFLEGSGGLFE 302
OsPFK02 DCCLIPESPFYLEGEGGLFE 371
OsPFK05 DCCLIPESPFYMDGEGGLLQ 373
AtPFK4 DCCLIPESPFFLEGKGGLFE 352
AtPFK2 DCCLIPEMDFYLEGKGGLFE 286
OsPFK03 DCCLIPEVDFYLEGKGGLFE 109
OsPFK09 DCCLIPEEDFYLRGAGGLFD 301
OsPFK07 DVCLIPEVPFTLDGEYGVLR 382
OsPFK08 DVCLIPEVSFTLDGEHGVMR 380
OsPFK10 DICLIPEVPFTLDGPNGVLQ 377
AtPFK5 DICLIPEVPFNLHGPNGVLK 390

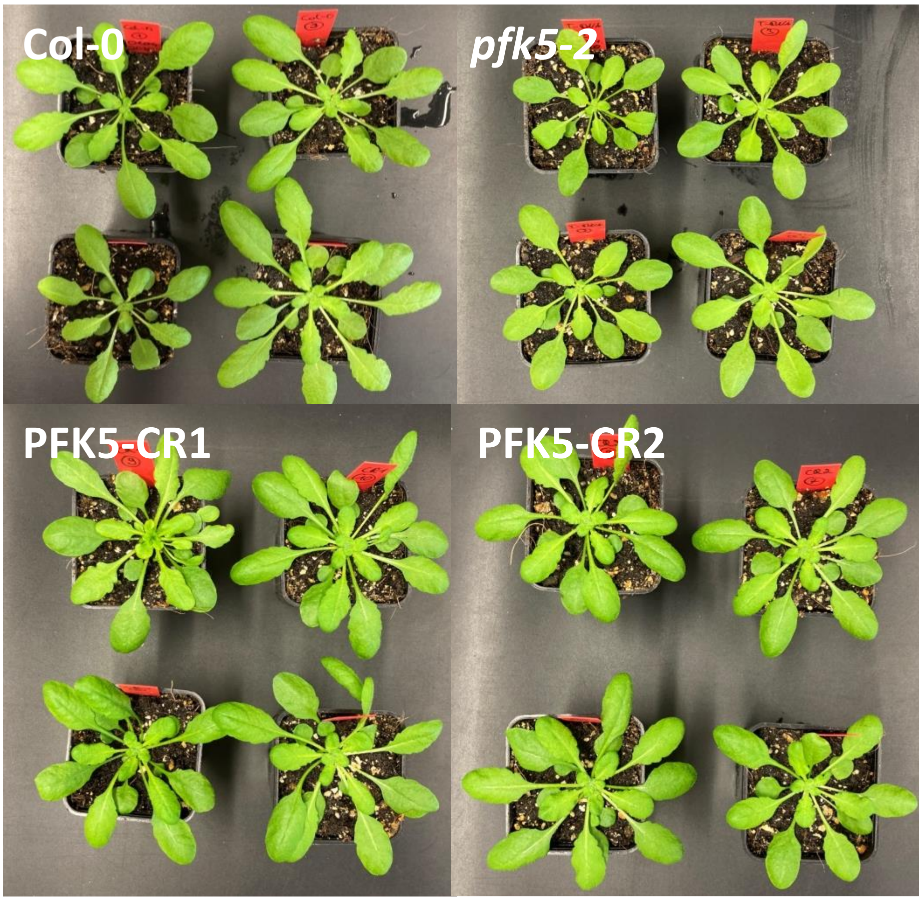

Supplementary Figure S5: Representative pictures of five-week-old *pfk5-CR1*, *pfk5-CR2*, *pfk5-2* and wildtype Col-0 plants grown on soil (representative pictures from the experiment in Figure 9C). One pot has the width of 5.3 cm.

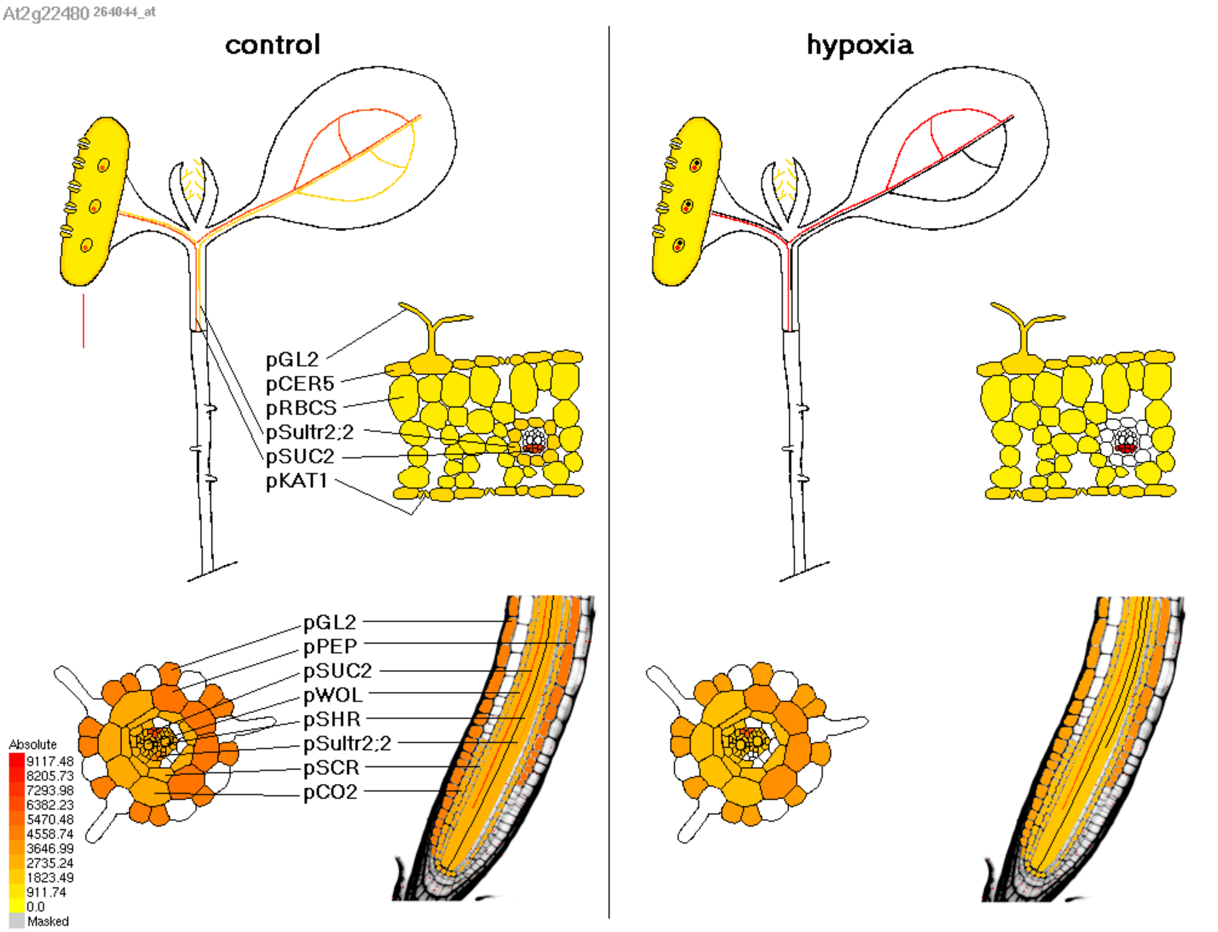

Supplementary Figure S6: Expression atlas of *AtPFK5* from the eFP browser (http://bar.utoronto.ca/, Winter et al. 2007, Mustroph et al. 2009).

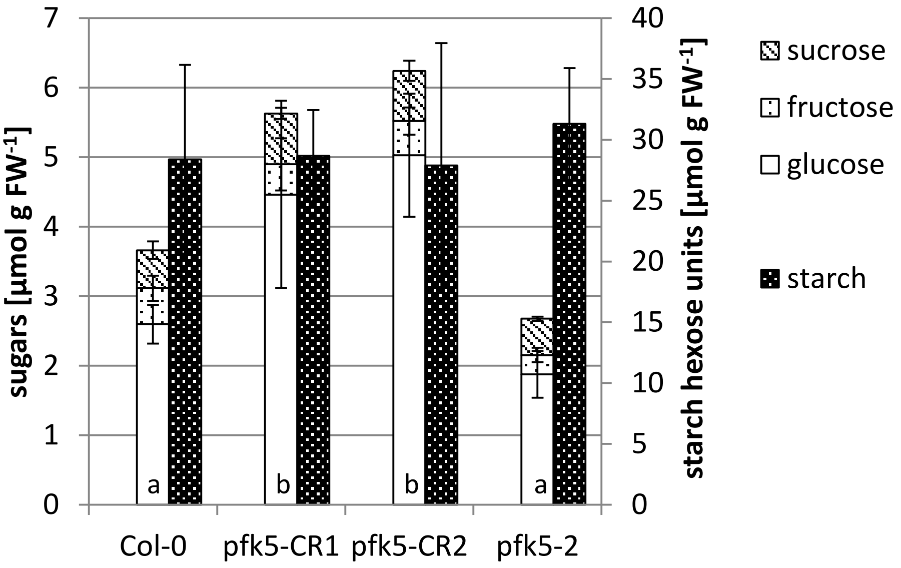

Supplementary Figure S7: Sugar content in leaves of five-week-old *pfk5-CR1*, *pfk5-CR2*, *pfk5-2* and wildtype Col-0 plants grown on soil, harvested at the end of the day. Data are means ± SD of n=4. Letters indicate significant differences at $p < 0.05$ for glucose content (ANOVA, Post-hoc Tukey test). Fructose, sucrose and starch contents were not significantly different between genotypes.

Supplementary Table S1 (separate Excel file): Primer sequences (a) and Protein IDs (b) used in this study

Supplementary References:

Huerta-Cepas J, Serra F, Bork P (2016) ETE 3: Reconstruction, Analysis, and Visualization of Phylogenomic Data. Mol Biol Evol 33: 1635-1638.

Mustroph A, Zanetti ME, Jang CJ, Holtan HE, Repetti PP, Galbraith DW, et al. (2009) Profiling translatomes of discrete cell populations resolves altered cellular priorities during hypoxia in Arabidopsis. Proc Natl Acad Sci USA 106: 18843-18848.

Winter D, Vinegar B, Nahal H, Ammar R, Wilson GV, Provart NJ (2007) An "Electronic Fluorescent Pictograph" browser for exploring and analyzing large-scale biological data sets. PLoS One 2: e718.
